# Patterns and distribution of de novo mutations in multiplex Middle Eastern families

**DOI:** 10.1038/s10038-022-01054-9

**Published:** 2022-06-20

**Authors:** Muhammad Kohailan, Waleed Aamer, Najeeb Syed, Sujitha Padmajeya, Sura Hussein, Amira Sayed, Jyothi Janardhanan, Sasirekha Palaniswamy, Nady El hajj, Ammira Al-Shabeeb Akil, Khalid A. Fakhro

**Affiliations:** 1 College of Health and Life Sciences, Hamad Bin Khalifa University, P.O. Box 34110 Doha, Qatar; 2 Department of Human Genetics, Sidra Medicine, P.O. Box 26999 Doha, Qatar; 3 Biomedical Informatics Division, Sidra Medicine, P.O. Box 26999 Doha, Qatar; 4 Department of Genetic Medicine, Weill-Cornell Medical College, P.O. Box 24144 Doha, Qatar

**Keywords:** Rare variants, DNA methylation

## Abstract

While de novo mutations (DNMs) are key to genetic diversity, they are also responsible for a high number of rare disorders. To date, no study has systematically examined the rate and distribution of DNMs in multiplex families in highly consanguineous populations. Leveraging WGS profiles of 645 individuals in 146 families, we implemented a combinatorial approach using 3 complementary tools for DNM discovery in 353 unique trio combinations. We found a total of 27,168 DNMs (median: 70 single-nucleotide and 6 insertion-deletions per individual). Phasing revealed around 80% of DNMs were paternal in origin. Notably, using whole-genome methylation data of spermatogonial stem cells, these DNMs were significantly more likely to occur at highly methylated CpGs (OR: 2.03; *p* value = 6.62 × 10^−11^). We then examined the effects of consanguinity and ethnicity on DNMs, and found that consanguinity does not seem to correlate with DNM rate, and special attention has to be considered while measuring such a correlation. Additionally, we found that Middle-Eastern families with Arab ancestry had fewer DNMs than African families, although not significant (*p* value = 0.16). Finally, for families with diseased probands, we examined the difference in DNM counts and putative impact across affected and unaffected siblings, but did not find significant differences between disease groups, likely owing to the enrichment for recessive disorders in this part of the world, or the small sample size per clinical condition. This study serves as a reference for DNM discovery in multiplex families from the globally under-represented populations of the Middle-East.

## Introduction

De novo mutations (DNMs) play major roles in organismal evolution, in which they are responsible for creating biological diversity [1]. Though rare, DNMs can also disrupt core developmental pathways, resulting in severe genetic disorders, such as autism spectrum disorder, congenital heart disease, and intellectual disability [2, 3], and could explain the recurrence of such severe disorders in outbred populations despite the detrimental impact on reproductive fitness.

As the interest in understanding the roles of DNMs grows, it has become useful to assess their pattern and distribution in both simplex and multiplex families from ancestries representing the diversity of global populations. The average DNM rate in humans is estimated to be around 1–1.3 × 10^−8^ mutations per base per generation [4–6]. However, estimates are somewhat complicated by the coverage efficiency in both parents and their children and by the genomic context, e.g., the higher mutation rates in GC-rich regions across different organisms, including humans [7, 8]. Moreover, considering the technical challenges produced by PCR bias or sequencing errors, and the relatively low number of DNMs in the genome, accurate calling and detection requires approaches that can yield the highest sensitivity without compromising specificity; and such combinatorial approaches must be developed using complementary tools that help increase the likelihood of capturing true positives while limiting erroneous calls.

While previous studies have looked at DNMs in different populations [4–6, 8–12], they have been somewhat limited by the use of separate parent-offspring trios or a small number of multi-generational families. Further, most studies to date have been performed in outbred populations, with inadequate representation of the highly consanguineous Middle Eastern cohorts. We thus aimed to explore the rate and distribution of single-gamete DNMs detected using short-read whole-genome sequencing (WGS) in a cohort of 146 multi-offspring families (353 unique trios) enrolled in a large pediatric tertiary care center in the Middle East. We applied three complementary tools to generate an integrated list of DNMs for every individual, which was used to estimate the DNM rate, determine the parent-of-origin, and investigate the impact of parental age on DNM count. We also examined the DNM mutational spectra and the distribution of DNMs through genome methylation maps for both gonadal and somatic tissues. Finally, we investigated the impact of consanguinity, ancestry, and disease status on DNM counts in our cohort. To the best of our knowledge, this is the first large-scale assessment of DNMs in a Middle Eastern multiplex family cohort, and it establishes a reference for this globally under-represented population.

## Materials and methods

### Sample collection and DNA extraction

We gathered 353 trios from a total of 146 multi-offspring families (Table 1) that were enrolled for research under institutional review board (IRB) protocols IRB#1610004943 and IRB#712017158 at Sidra Medicine. Written informed consent was obtained from each study subject. Whole blood samples were collected and total genomic DNA was extracted from each sample using DNeasy Blood & Tissue Kit (Qiagen sciences LLC, Germantown, MD, USA), and 1500 ng was used for WGS.

**Table 1:** Description of the included families and identified DNMs in the study cohort

Description	Count
Total cohort size	645 samples
Trios (males, females)	353 (190, 163)
Phenotypes	
Neurogenetic	92
Craniofacial	17
Endocrine	9
Multi-system	17
Other	25
Healthy	193
Sub-populations	
African	33
South-Asian	67
Middle-Eastern	207
Caucasian	21
Other	25
Total families	146
Consanguineous families	47
Median fathers’ age	34 years old
Median mothers’ age	29 years old
Total identified de novo variants	27,168
SNVs (median per individual)	24,808 (70)
INDELs (median per individual)	2360 (6)
Effective genome coverage	2.797 × 10^9^
SNVs rate	1.25 × 10^–8^
INDELs rate	1.07 × 10^−9^

### Whole-genome sequencing and quality check

Libraries were prepared using TruSeq DNA Nano kit (Illumina Inc, San Diego, CA, USA), and samples were sequenced to an average depth of 30X using Illumina HiSeq X at the Core Genomics Lab at Sidra Medicine. Raw reads were aligned to GRCh37 using the standard settings of the BWA kit v0.7.15 [13]. Pre- and post-alignment quality checks were performed using FastQC v0.11.2 [14] and Picard v2.17.6 [15]. The heterozygosity and missingness rate were plotted after variant calling to evaluate the sample quality. We also performed a sex check, and we removed samples that shared a lot of variants, which implies contamination has occurred.

### De novo variant identification and pipeline optimization

To optimize de novo variant detection, we used three tools combined with a manual inspection of a random set of ~3500 de novo variants by integrative genomics viewer (IGV) to optimize the pipeline’s sensitivity and specificity for each tool as follows: First, the VCF file generated by FreeBayes v1.1.0 [16] was manually filtered for de novo variants based on genotype, alternate allele ratio (0.25 to 0.75 in proband, and 0 in all other family members), read depth (≥12), and quality score (≥30 for single-nucleotide variants [SNVs] and ≥80 for insertions-deletions [INDELs]). Second, VarScan v2.3.9 [17] was used to call variants directly from each trio’s mpileup file generated using SAMTools v1.9 [18]. Filtration was based on the genotype, alternative allele depth (0 in parents), “DENOVO” and “PASS” tags, *p* value (≤0.005), and allelic ratio (Freq; ≥0.25 for SNVs and ≥0.30 for INDELs). Third, we used a reference-independent k-mer-based variant caller, i.e., RUFUS v1.0 [19], to call variants directly from BAM files. We used the recommended k-mer size (25 bases) and kept variants tagged as “DeNovo”.

After calling, we combined the three lists of variants for each individual and marked variants seen by two or three tools as “pass”, while those unique to only one tool underwent processing with more stringent filtration thresholds (FreeBayes: depth ≥14, RUFUS: Qual ≥16 for SNVs and ≥17 for INDELs, and VarScan: Freq ≥0.3 for SNVs and ≥0.35 for INDELs) to be approved or excluded. As a final step, we annotated variants using SnpEff 4.3 T [20] to add information on the predicted consequences of the variants, evolutionary conservation, population frequency, clinical disease associations, etc. To remove population-specific rare variants missed in the parents, we also filtered out variants with an allele frequency of >0.1% in different databases [21–27].

### Calculating effective genome coverage

The effective number of bases covered by WGS was calculated as previously described [4]. The average initial number of bases covered was 2.84 billion and 56.07 million for non-CpGs and CpGs, respectively. After filtration, 2.74 billion non-CpGs and 53.66 million CpGs remained; giving an average total of 2,796,691,061 bases.

### Calculating DNM base-substitution frequencies

For the DNM spectra, we merged substitutions that represented the same event on complementary strands (e.g., C > T was considered the same as G > A) and calculated the fraction of each possible type. For the mutational signature, we extracted the DNA sequence triplet around each variant from the GRCh37 reference genome using the “getfasta” module from bedtools [28]. We then calculated the proportion of each of the resulting 96 triplets compared to the total number of DNMs.

### Calculating GC content around DNMs

To calculate the percentage of GC content around de novo SNVs and INDELs, we first determined the regions flanking DNM sites in sliding windows ranging from 10–1000 bases. We then extracted these regions from the GRCh37 reference genome using BEDTools v2.28 [28] and calculated the GC content fraction within each window of bases.

### Determining the parent-of-origin for de novo mutations (phasing)

We followed a read-based phasing approach to phase the de novo variants. This approach requires the existence of an “informative” inherited heterozygous variant that can be phased to a parent and is in the same sequencing read as the DNM, allowing the DNM to be phased. We used Unfazed v0.2.3 for this purpose [29].

### Effect of parental age on DNM count

To measure the effect across all families, we calculated the fraction of paternal/maternal DNMs within the total number of phased DNMs in each proband, scaled this to the entire DNM count per individual, and plotted these against parental age at conception. We used a Poisson regression model (using the glm function with the option link = “identity”) to examine the relationship between paternal age and DNM count, following the same style as published before [30]. To measure the effect *within* each family, we ran the analysis on families with four or more children.

### Calculating relatedness scores

To perform consanguinity analysis, we calculated the relatedness scores using relatedness2, part of the KING inference method in VCFtools [31]. The relatedness score, or kinship coefficient (PHI), is defined here as the probability of finding identical alleles when randomly selecting one allele from each individual [32]. We used the recommended cutoffs of these scores to distinguish between 1^st^ degree cousins (<0.177, ≥0.0884), 2^nd^ degree cousins (<0.0884, ≥0.0442), and unrelated parents (<0.0442).

### Correlation between local DNM rate and rates of methylation

We first downloaded a bigWig whole-genome bisulfite sequencing profile of human adult spermatogonial stem cells (SSCs) [33], then determined all CpG positions in the genome using the FASTA reference genome (GRCh37) and appended the SSCs’ methylation values to these positions. We also extracted the SSCs’ methylation values for our list of DNMs that occurred at CpG sites. Afterward, we counted the number of sites with high (>50%) and low (≤50%) degrees of methylation for both lists. We next calculated the fraction of DNM-CpGs of the whole-genome CpGs in each methylation interval and measured the fold difference between these fractions. We also downloaded the methylation profiles of human liver cells and peripheral blood mononuclear cells (PBMCs) to use as controls [34, 35].

### Statistical analysis

All statistical analyses were performed in R statistical language (v3.4.3). For scatter plots, Pearson correlation coefficients were calculated. *p* values in all boxplots were calculated using pairwise Student’s t-test. In the methylation analysis, binomial *p* values were used to calculate the significance of the fold difference between fractions of methylation levels. *p* values of less than 0.05 were considered statistically significant.

## Results

### Cohort description and QC

A total of 146 families (*n* = 645 individuals) were enrolled in this study, of which 47 (32%) reported a history of consanguinity (first- or second-degree parental relatedness). Parental ages at conception varied as follows: fathers (median: 34 years old, range: 21–50) and mothers [29, 16–44]. Family sizes differed across the cohort (median: 2 offspring, range: 1–10), with more than 70% of families being multi-offspring (Supplementary Fig. 1). All participants underwent WGS to an average depth of 31.6X (Supplementary Fig. 2), with variants aligned and called as described in the Methods. After QC, a total of 353 unique trios could be established from the cohort (one child plus both parents), which were selected for DNM calling and annotation. The trios included 190 males and 163 females, 45.3% of whom had an underlying rare disorder. A summary of these statistics is provided in (Table 1).

### A combinatorial approach to calling DNMs and calculating DNM rate

To ensure the comprehensive ascertainment of variants and to improve sensitivity and specificity, we used a combination of three different approaches to identify de novo mutations with a three-step workflow (details in Methods, Supplementary Fig. 3). In total, we identified 24,808 high-quality de novo single-nucleotide variants (SNVs) and 2360 INDELs in 353 trios (Table 1 and Supplementary Fig. 4), with the median genome containing 70 de novo SNVs (average = 70.3) and 6 de novo INDELs (average = 6.7). Taking into consideration the effective genome coverage of around 2.797 billion base pairs (see Methods) and genomic diploidy, we calculated a median DNM rate of 1.25 × 10^−8^ and 1.07 × 10^−9^ per base per generation for SNVs and INDELs, respectively. These rates are consistent with previous reports [4, 5, 30, 36].

### Effect of parental age on DNM count and differences across families

To determine the parental contribution to DNMs, we sought to determine the parent-of-origin where possible using read-based phasing. Given the 150-bp read length, we were able to phase 13% (range: 4.2–25%) of the de novo variants on average (Supplementary Fig. 5). Among the 3537 variants phased, 2817 were paternal in origin and 720 were maternal. This corresponded to a paternal-to-maternal DNM phasing ratio of ~3.91:1, in line with prior estimates [30].

**Fig. 1:**
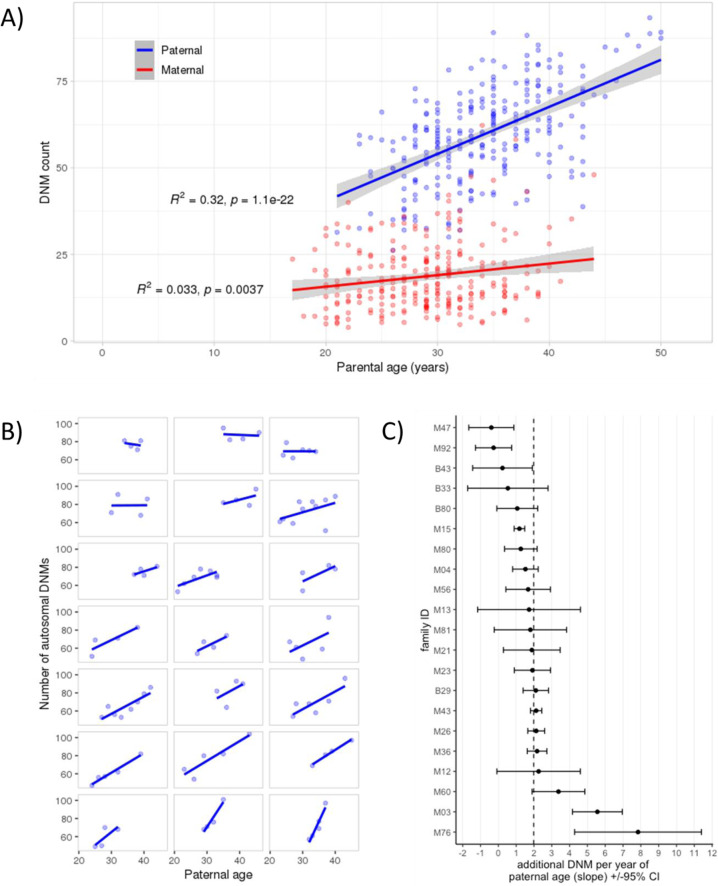
Parental age effects on DNM counts. **A** Correlation between parental age at conception and number of phased DNMs normalized to the total number of phased DNMs in each individual, performed across all families. The blue regression line (slope = 1.36, 95% CI = 1.11–1.61) shows paternally phased DNMs, while the red line (slope = 0.33, 95% CI = 0.11–0.56) shows maternally phased DNMs. **B** Paternal age is plotted against the number of total autosomal DNMs for individuals in large families (number of offspring ≥4, total = 21 families), with each family analyzed separately. Families were plotted in order of ascending correlation for easier visualization. Slopes of the regression lines range from −0.54 (95% CI: − 7.06–5.97) to +7.74 (95% CI: 3.14–12.33). **C** A Poisson regression for each large family. The plot shows the slope of each regression ± 95% confidence intervals. The vertical line indicates the average paternal age effect for all families in this model

We then checked for correlations between parental age and DNMs across the cohort (Fig. 1A). Although we observed a significant increase in DNMs by 1.36 per year of paternal age (Pearson correlation; 95% CI: 1.11–1.61, *p* = 1 × 10^−22^), we observed a weak correlation with maternal age, with an increase of 0.33 DNMs per year (Pearson correlation; 95% CI: 0.11–0.56, *p* = 3.8 × 10^−3^). These results agree with previous findings that show parental age effects on the DNMs found in offspring [5, 6, 9, 37, 38].

Given that DNMs count can be affected, in addition to paternal age, by other factors such as family membership, number of offspring, and ancestral population, we applied a Poisson regression analysis that integrates these factors into the model. Initially, we built a null model focusing on the paternal age effect on the DNM count but using only families with more than three offspring (*n* = 21). This showed an estimated paternal age effect of 1.57 DNMs per year (95% CI: 1.29–1.85, *p* < 2.2e−16) (Supplementary Table 1). Then we fitted a Poisson regression model by adding family membership to the model that incorporated paternal age and DNM count, and found that the paternal age effect significantly varies between families, ranging from −0.39 (95% CI: −1.66–0.87) to 7.8 (95% CI: 4.28–11.40) additional DNMs per year (Fig. 1B, C). This interaction model fits better than the null model and gives an improved regression model (*p* = 0.002). This model shows an average increase of 2.1 (95% CI: 1–3.2) DNMs per year of paternal age. We also examined two more Poisson models that test the paternal age effect on DNM counts and separately add number of offspring and population, but both factors had no significant effect on the relationship of paternal age and DNM count (Supplementary Table 1).

### Effect of additional siblings on DNM detection accuracy

**Fig. 2:**
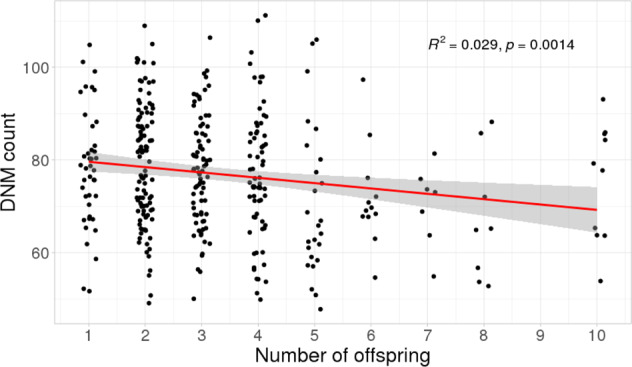
Effect of family size on DNM counts. Number of offspring per family is plotted against DNM count in individuals. The red line represents the regression line (slope = −1.15) with 95% confidence intervals shown in gray

We stratified the cohort based on the number of offspring per family to investigate if additional offspring reduced the DNM counts in each “index” child (Supplementary Fig. 1). We found the average number of DNMs per individual to be lower in larger families compared to smaller families, with a reduction of around 1.15 DNMs per added sibling (Fig. 2), suggesting that sequencing additional family members can significantly improve the ability to discriminate true de novo variants from rare inherited ones.

### De novo mutation load and consanguinity

**Fig. 3:**
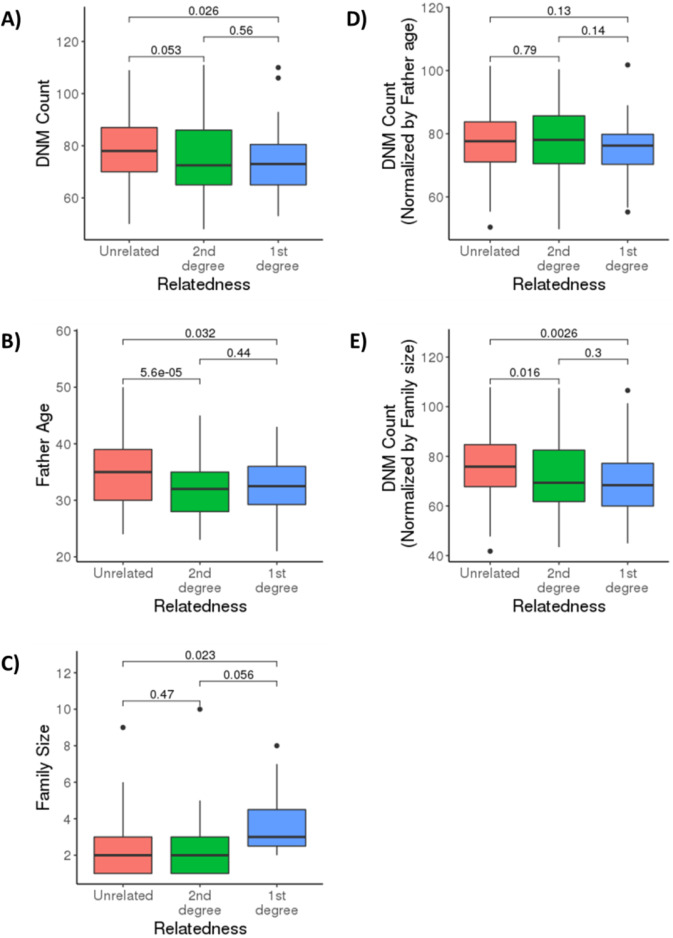
De novo mutation load and consanguinity. Parents in each trio in the dataset were categorized into 1st degree cousins (blue), 2nd degree cousins (green), and unrelated (red). Boxplots show the median and interquartile range, and *p* values are shown above brackets. Plots show the correlation between relatedness scores and (**A**) DNM count, (**B**) father’s age at conception, (**C**) family size, (**D**) DNM count after correcting for father’s age, and (**E**) DNM count after correcting for family size

Given the high level of consanguinity (~32%) in our cohort, we explored whether there was evidence for a correlation between consanguinity and DNM count (Fig. 3, Supplementary Fig. 6). Rather than relying solely on reported parental consanguinity, we computed each child’s relatedness score using KING (see methods) [31]. Although the number of DNMs was not expected to be affected by consanguinity, the offspring of consanguineous marriages appeared to have fewer DNMs (*p* value = 0.033) (Fig. 3A). To rule out confounders impacting this correlation, we also examined the relationship between relatedness score and both the father’s age at conception (Fig. 3B) and family size (Fig. 3C). Notably, consanguineous parents in our cohort appeared to have had children at younger ages than non-consanguineous parents, as well as larger family sizes, which, as explained earlier, reduces the DNM count due to sibling sharing. As expected, correcting for these factors (the father’s age in particular) uncoupled the relatedness score from the DNM counts (Fig. 3D, E), providing a rational explanation for why the offspring of consanguineous parents appeared to have fewer DNMs than non-consanguineous trios.

### DNM spectra and mutational signature

We next examined the distribution of DNMs in relation to base changes (Supplementary Fig. 7A). Consistent with previous reports [39], we found a nearly 2-fold enrichment in transitions versus transversions, with 35% of DNMs being C > T. To drill deeper into the genomic context of the DNMs, we examined all possible DNA sequence triplets at the DNM sites which, together, make up the mutational signature of de novo mutations (Supplementary Fig. 8). Among the highest proportion of DNMs (i.e., C > T substitutions), CpG sites were found to contribute to a large fraction of the DNM events. The same mutational signature was also discovered previously in three different trio datasets [40]. We then followed the above approach to compare the fractions of phased DNMs from both parents (Supplementary Fig. 7B). We found no statistically significant difference in the DNM spectra by parent-of-origin, likely due to the relatively limited number of phased DNMs per individual.

We further examined the effect of local GC content on mutability. Using a sliding window approach (with windows ranging from 10–1000 bases), we extracted the genomic sequence from around each DNM from the GRCh37 reference genome and calculated the GC content surrounding SNVs or INDELs (Supplementary Fig. 9). We found a higher GC content near SNVs and a lower content near INDELs compared to the average genomic GC content of 41% [41].

We next calculated the mutation rates of both transition and transversion variants with respect to CpG site (Supplementary Table 2). We found that CpG dinucleotides had much higher mutation rates compared to non-CpGs, and the difference was clear.

### CpG methylation as a driver for DNM development

The high correlation between DNMs and CpG sites suggested that methylation levels play a role in the genesis of DNMs in parental gametes. To assess this, we compared the mutation rates at CpG sites with respect to the level of methylation (i.e., percentage of reads containing a methyl group) across human tissues. First, when we examined adult spermatogonial stem cells (SSCs) [33], we observed a total of 3,801 variants in our DNM catalog at CpG sites, 475 of which were paternal in origin. Surprisingly, for the paternally phased variants, we found that the highly methylated CpG sites (i.e., >50% of reads methylated) were 2.03 times (binomial *p* value = 6.62 × 10^−11^) more likely to have DNMs than the low-methylation sites (Table 2). To improve the specificity of this observation, we performed the same analysis using the methylation profiles of two other human tissues as controls: liver cells and PBMCs [34, 35]. We found much smaller fold-change differences between the methylation levels in terms of mutation rate (binomial *p* values = 0.03 and 0.004 for liver cells and PBMCs, respectively). These results provide further evidence for the key role of CpG methylation in the development of de novo mutations.

**Table 2:** Fold difference in the fraction of DNMs based on methylation levels

Cell type	Methylation level	DNM CpGs	All CpGs	Fraction	Fold difference	*P* value (Binomial)
SSCs	≤50%	86	8,804,182	9.77 × 10^-6^	–	–
	>50%	389	19,617,481	1.98 × 10^-5^	2.03	6.62 × 10^−11^
Liver cells	≤50%	288	18,383,721	1.57 × 10^−5^	–	–
	>50%	187	10,037,942	1.86 × 10^−5^	1.19	0.03
PBMCs	≤50%	316	20,440,889	1.55 × 10^−5^	–	–
	>50%	159	7,980,774	1.99 × 10^−5^	1.29	0.004

### DNM localization and count in different populations and disease phenotypes

We next examined the genomic localization of the 27,168 DNMs in our cohort. Among these variants, 459 were in coding regions (average per child = 1.3, median = 1). This represents 1.7% of the total number of variants, which is consistent with the proportion of coding bases in the human genome. We also found 43 (0.16 %) loss-of-function de novo variants, of which 13 (30%) were predicted to cause nonsense mediated decay.

**Fig. 4:**
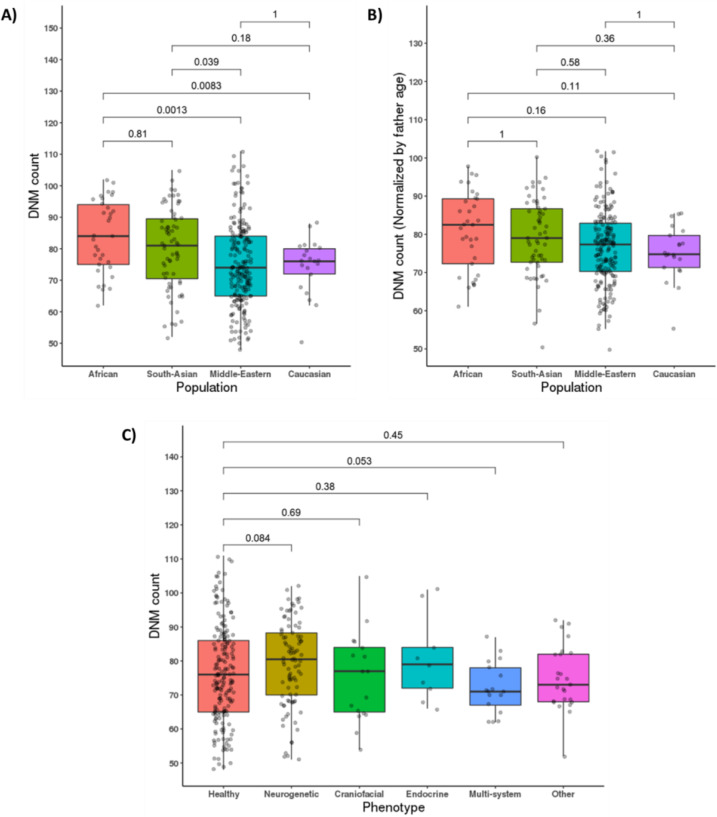
DNM counts by sub-population and disease phenotypes. Boxplots show the median and interquartile range, and *p* values (Bonferroni corrected in **A** and **B**) are shown above brackets. Plots show the (**A**) DNM counts in different populations, (**B**) DNM counts in the populations normalized to the father’s age, and (**C**) DNM counts with regard to disease phenotypes

We stratified our cohort based on ethnicity and disease phenotype to assess if there were differences in the DNM counts in these categories. African and South-Asian populations seemed to have a significantly higher number of DNMs compared to Middle Eastern and Caucasian populations, as shown in Fig. 4A. However, when we accounted for differences in paternal age between these populations (Fig. 4B), we found that the fathers’ ages at conception could be the factor driving the differences in DNM counts across the populations. After correcting for the father’s age, none of the populations remained significantly different from the others, although the African population showed a higher trend across populations.

After looking into the differences among phenotypes in terms of DNM counts, we found that probands with neurogenetic disorders had, on average, more DNMs compared to healthy subjects, although that difference did not achieve statistical significance. We also found no significant differences between any of the other phenotype groups and healthy subjects (Fig. 4C). To delve deeper into the variants in our dataset, we used the annotated files to compare several metrics between the different phenotypes. We compared the percentage of variants with certain thresholds of combined annotation-dependent depletion (CADD) scores (Supplementary Fig. 10A), genomic evolutionary rate profiling (GERP) scores (Supplementary Fig. 10B), combined CADD and GERP scores (Supplementary Fig. 10C), and predicted loss-of-function intolerance (pLI) scores (Supplementary Fig. 10D), with no significant differences between the phenotypes.

Finally, we tested if the variants with different functional effects were enriched in certain phenotypes (Supplementary Fig. 11). To do this, we first compared the phenotypes using the normal functional impact annotation of “LOW”, “MODERATE”, and “HIGH” (Supplementary Fig. 11A). We then sub-categorized the variants, based on their functional impact, into protein non-disrupting (“MODIFIER” + “LOW”) and protein-disrupting (“MODERATE” + “HIGH”) variants (Supplementary Fig. 11B). We also failed to find any significant differences between the DNM distributions across the different phenotype groups in this regard.

## Discussion

In this study, we investigated the spectrum and distribution of DNMs in 146 multiplex families from the genetically under-represented population of the Middle East. We devised a highly sensitive and specific combinatorial approach for DNMs calling and discovered 27,168 high confidence DNMs in 353 unique individuals, with a median of 70 de novo SNVs and 6 de novo INDELs per genome, consistent with previous estimates [4, 5, 30, 36]. We noted that while each tool independently gave similar numbers of DNMs per individual, the advantages of a combinatorial approach over single tools lies in establishing consensus to remove false positives and rescue false negatives, leading to higher accuracy and validation of DNMs.

Selection of the candidate DNM calling tools was mainly driven by our aim to use approaches that rely on different underlying algorithms to maximize the sensitivity and specificity of the pipeline. After investigating many tools for de novo calling, we selected the following three: FreeBayes, VarScan and RUFUS. FreeBayes uses a haplotype-based approach to call variants [16]. This method is relatively more comprehensive, compared to alignment-based methods [18, 42, 43], in that it relies on the actual sequences of reads aligned to a particular target, rather than only their alignment, allowing more sensitive detection of variants at regions with highly similar sequences. However, FreeBayes and many other tools apply probabilistic algorithms to call variants and examine their confidence, which can be affected by several confounders, including read depth. VarScan employs a heuristic variant calling approach that depends on meeting certain threshold settings for read depth and other parameters [17]. RUFUS, meanwhile, employs a reference-independent approach that directly compares raw sequence data of the samples to be assessed, with greatly increased specificity [19].Thus, these 3 tools are complementary in their approaches, and help overcome certain issues such as repetitive sequences, variable depth of coverage, and reference bias, while maintaining reasonable resource utilization and speed of calling.In order to establish parent of origin effects, we phased the DNMs using a read-based approach, relying on neighboring heterozygous “informative alleles” to unambiguously assign alleles to either parent [44]. In this way, we were able to phase ~13% of all variants, of which around 80% appeared to be paternal in origin. Although we had a relatively low phasing rate in the study cohort, our results correspond with previously reported findings which showed similar proportions of parental gamete-of-origin [4, 30]. These observations, together with the hypothesis that single-gamete DNMs arise during genome replications of the parental gamete [44], underscore the role of spermatogenesis in DNM development.

In line with previous studies [5, 6, 9, 37, 38], we found the number of DNMs to be increased with advancing parental age, and the rate was different for fathers versus mothers. It has been hypothesized that the accumulation of DNMs observed with advancing paternal age arises from incidental copying errors during genome replication over the course of spermatogonia mitosis [38, 44].

Interestingly, significant inter-family variability (*p* = 0.001) was observed when assessing the effect of paternal age on DNM accumulation. This variability is unlikely to be driven by differences in family size (Supplementary Table 1). However, three potential reasons could explain this variation. First, the overall mutation rate might have been affected by differences in the genetic make-up of the families and their environmental exposure. Second, the age at puberty of the parents, at which gametogenesis starts, may have differed, and thus a parent that experienced a late puberty would accumulate fewer mutations than a father with the same age of conception who underwent puberty earlier. Third, although replication errors have been suggested to be the main contributor to DNM development, other sources (e.g., DNA damage) could influence this variation and may have differed among families in our cohort [30, 45].

As part of our DNM calling pipeline to reduce the number of missed parental heterozygotes, we excluded variants shared between the probands and siblings in each family. One consequence of this was that variability in the number of siblings across families may have affected the DNM counts. To test this, we stratified the cohort based on the number of offspring per family and found that the average number of DNMs per individual decreased with an increase in the number of siblings, reflecting the importance of sequencing more siblings in rare disease families where the proband is suspected to have pathogenic DNMs.

Furthermore, we explored if DNM rates in any way correlate with consanguinity, although such correlation is not actually expected in single-gamete DNMs, because they arise before zygote fertilization [30], and are thus independent of parent relatedness. In our study cohort, we observed a nominally significant correlation; however, this correlation appeared to be confounded by both the father’s age at conception (consanguineous couples in our cohort conceived earlier) and the availability of more siblings in consanguineous families (larger kindreds). This observation sets a valuable precedent for studies in similar population settings, where such variants ought to be taken into consideration to avoid confounded results and interpretations.

We next examined the distribution of DNMs by substitution type and found a marked enrichment in transitions over transversions. Although the mutational spectrum was previously shown to be different in terms of the parent-of-origin [30], we found no significant differences, which is likely due to the small number of phased variants in our cohort. Furthermore, by examining the mutational signatures of the DNMs, we found that CpG sites disproportionately contributed to DNM events, which was also seen in other recently examined datasets [40]. Moreover, we found a higher GC content near SNVs (average of 44.2% per 10 bp window), compared to the average genomic GC content of 41% [41]. This was expected, as a high GC content has been shown to affect the repair pathways and elevate the mutation rate [7, 46]. On the other hand, INDELs appeared to occur more often within lower GC content regions, which contradicts a previous study that showed a positive correlation between INDEL and GC content [47]. However, this contrary finding was not statistically significant, probably due to the cohort size.

We next questioned whether CpG methylation is a driving factor in DNM development. To investigate this in our cohort, we compared the DNM sites in the whole-genome methylation profiles of human adult SSCs to those of PBMCs and liver cells [33]. Indeed, highly methylated CpG sites were twice as likely to be mutated than low-methylation sites. Previous study on CpG substitution rate in introns of human genes have also shown a positive correlation [48]. Another study performed on methylation profiles generated by reduced representation bisulfite sequencing also showed that methylated CpG sites are relatively more likely to mutate than unmethylated CpGs [5]. This suggests that DNA methylation during spermatogenesis is a key DNM developmental mechanism.

We then stratified our cohort based on ethnicity and disease phenotype to assess differences in the DNM rates across these groups. In terms of population structure, the African population appeared to have more DNMs (although not significant) than other ethnicities, even after correcting for parental age at conception. This pattern could either have been confounded by factors not included in our calculations, as was seen in a previous study in which temperature was shown to affect mutation rates [49], or be a true population-specific pattern that contradicts a previous estimate which showed similar DNM rates among different sub-populations except for a reduction in the Amish population [1]. It could also be a consequence of the small sample size provided by the cohort. In terms of disease phenotype, many studies in the past have established a prominent role for DNMs in certain congenital and developmental conditions, e.g., congenital heart disease, intellectual disability, and autism [50, 51]. Here, we found no significant differences in DNM count or predicted impact/severity of DNMs in children with these conditions versus their control siblings, or for other disease categories represented across our cohort. There are several explanations for this. First, the cohort size within each disease category may have been too underpowered to detect significant differences in pairwise comparisons; however, even when we amalgamated several disorders together to compare against controls, the resulting differences by count and by predicted impact did not reach statistical significance. Second, given our families inhabit an area of the world with high consanguinity, it is possible that the allelic architecture driving disease in the affected probands is largely recessive, rather than de novo. Consanguineous families are known to be rich in homozygous recessive alleles that could be disease-causatives [52]. This would explain the overall equality of the burden of DNMs across siblings, despite disease status, which has important implications for studies looking at pediatric disorders in Middle Eastern settings. In particular, diseases long thought to be largely dominant (caused by DNMs) in global (outbred) cohorts may, in fact, involve recessive genes that have yet to be discovered in populations with high degrees of consanguinity [53–55]. Congenital heart disease is an example where causative mutations in *MCTP2* gene are known to be only dominant, but found to be recessive in a consanguineous cohort [56]. This has implications for the biomedical discovery of pathways that can be targeted for drug development and intervention. Importantly, this could lead to improvements in newborn and prenatal screening programs in regional populations to help with the early detection and possible eradication of conditions in the future. Third, we filtered our data for DNMs only existing in the probands and absent from both parents and siblings, which most likely removed not only the missed parental heterozygotes but also gonosomal and post-primordial germ-cell-specification variants that can appear in multiple children of the same parents. These post-zygotic variants were hypothesized to be correlated with different phenotypes, including adult-onset neurodegenerative disorders [57, 58]. Such an evidence of the role of these mutations is disease etiology might be sufficient to promote a difference between the phenotype groups.

Overall, the study produced a detailed illustration of DNMs in a large cohort of Middle Eastern families from Qatar. Because our cohort consisted of two-generation families only, we could not estimate the missed heterozygosity rate (variants wrongly genotyped as absent from a parent due to technical or computational errors but observed in a grandparent). We were also limited in our ability to identify the parent-of-origin for many DNMs due to the limited number of informative sites within short-read data. Nevertheless, most of our findings corresponded to globally observed rates and patterns of DNMs, thus establishing an important baseline dataset for Arab populations of the Middle East. Furthermore, while we were able to dissect the correlation between consanguinity and DNMs and show the impact of sequencing additional siblings on improving specificity, we were unable to replicate the differences in DNM rates between disease and control individuals across multiple diseases, nor across different predicted impact categories of DNMs in health and disease. This may be due to the small size per disease cohort, or to the effect of consanguinity enriching for recessive subtypes of clinical conditions rather than those caused by DNMs. Future studies with larger cohorts from the region will be required to resolve these discrepancies, with important implications for screening and intervention strategies in the future.

## Supplementary information

Supplementary file 1

Supplementary file 2
