# Supplementary material for: Patterns and distribution of de novo mutations in multiplex Middle Eastern families
Source: J Hum Genet. 2022 Jun 20;67(10):579–88. doi: 10.1038/s10038-022-01054-9 (PMC9510050; doi:10.1038/s10038-022-01054-9)
Supplement: Supplementary file 1 [file 10038_2022_1054_MOESM1_ESM.pdf]

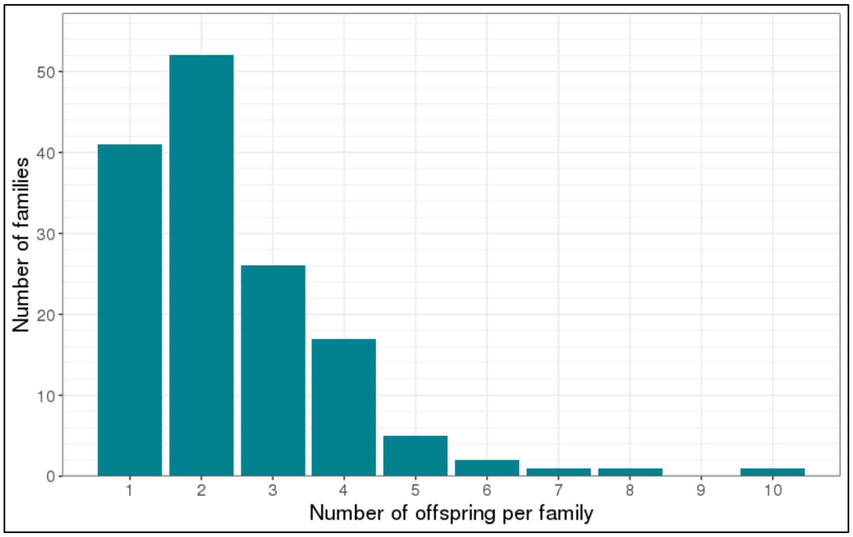

Supplementary Figure 1: Number of offspring per family. Plot shows family sizes and number of families in each size category.

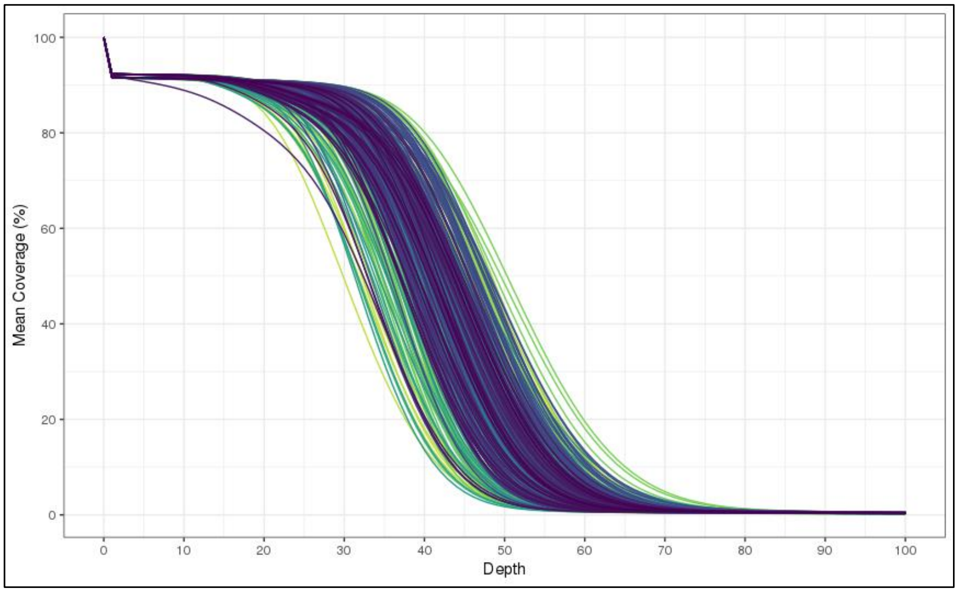

Supplementary Figure 2: Genome coverage. Percentage of genome covered at each specified depth.

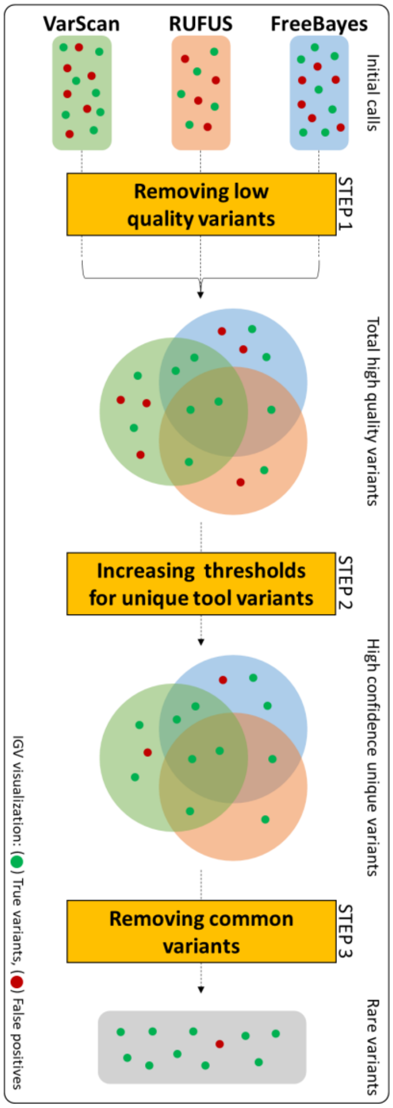

Supplementary Figure 3: Schematic overview of DNM calling pipeline used in this study. A combinatorial approach consisting of 3 different tools was applied to each "trio" to identify putative DNMs, and variants were manually inspected in IGV and scored. Quality filters were then applied per tool to eliminate possibly spurious calls. Variants unique to each tool underwent stringent filtration. Finally, all common variants were eliminated from the data. See Methods for more details on the above.

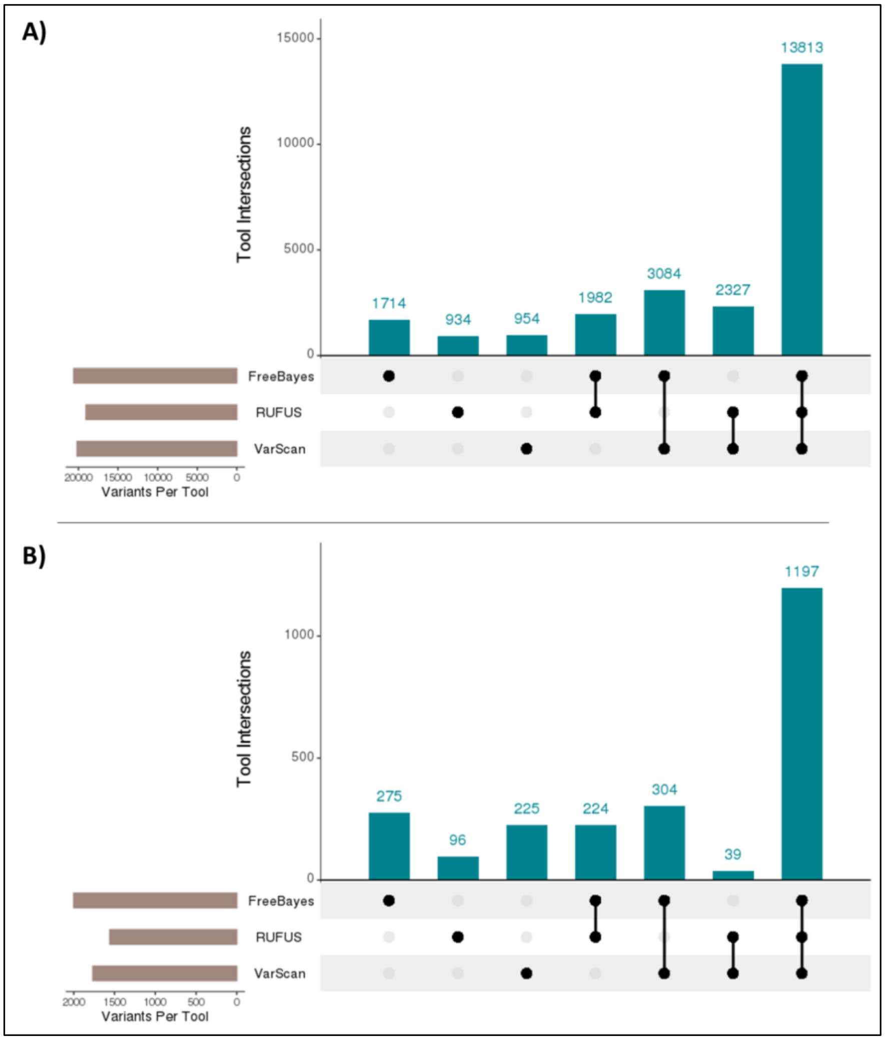

Supplementary Figure 4: UpSetR plot. Plot showing the overlap of variants by the tools used to detect them. (A) SNVs and (B) INDELs.

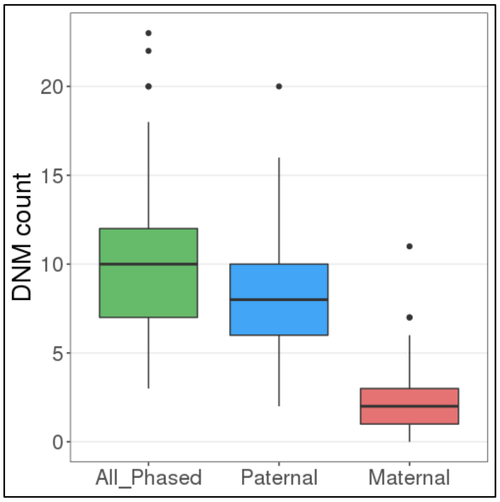

Supplementary Figure 5: Parent-of-origin determination for DNMs. Boxplots show the median and interquartile range. Total number of phased DNMs per individual is shown in green. The blue box shows the number of DNMs phased to the father. The red box shows the number of maternally phased DNMs.

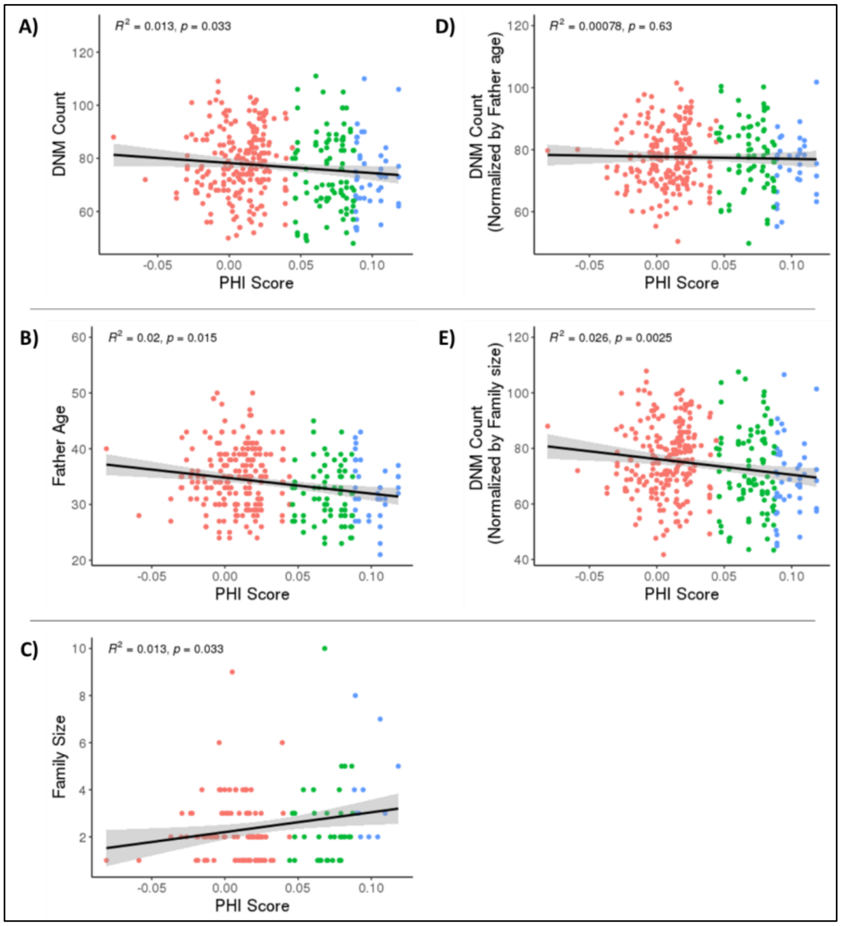

Supplementary Figure 6: *De novo* mutation load and consanguinity. Parents in each trio in the dataset were categorized into 1st degree cousins (blue), 2nd degree cousins (green), and unrelated (red). Plots show the correlation between relatedness scores and (A) DNM count, (B) father's age at conception, (C) family size, (D) DNM count after correcting for father's age, and (E) DNM count after correcting for family size. PHI scores represent the relatedness coefficients.

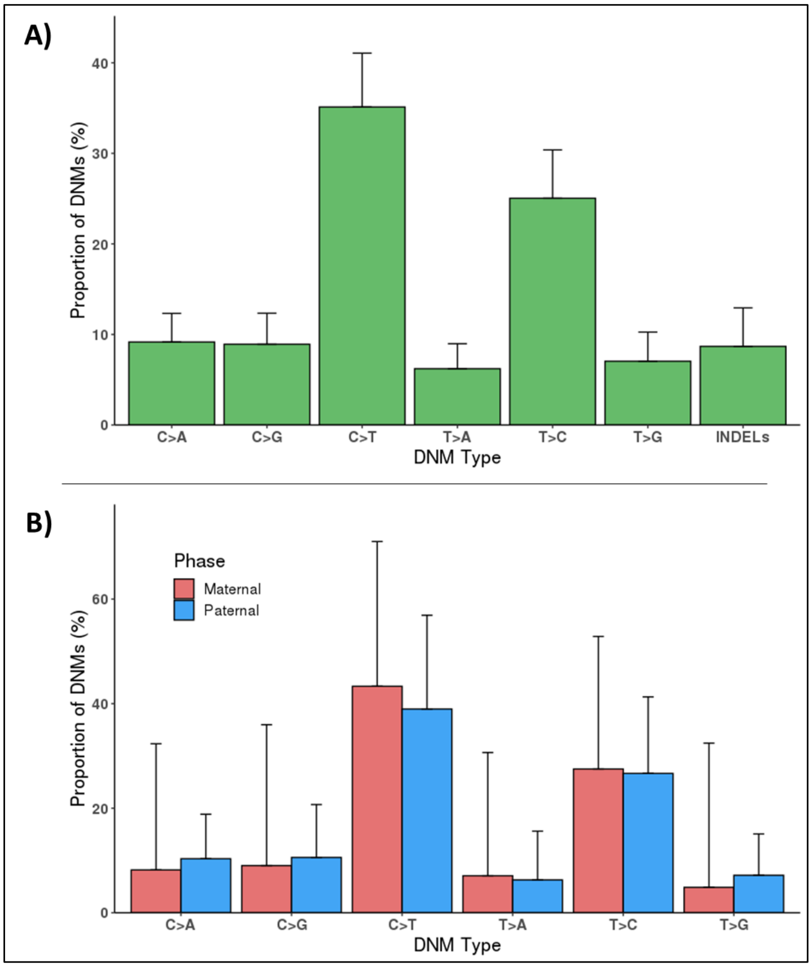

Supplementary Figure 7: *De novo* mutation spectra. (A) Proportion of each of the seven mutation categories to the total number of DNMs (n = 27,168). (B) Proportion as a function of paternally phased (n = 2,817), and maternally phased (n = 720) DNMs. All phased DNMs were SNVs. Data are represented as the mean ± SEM.

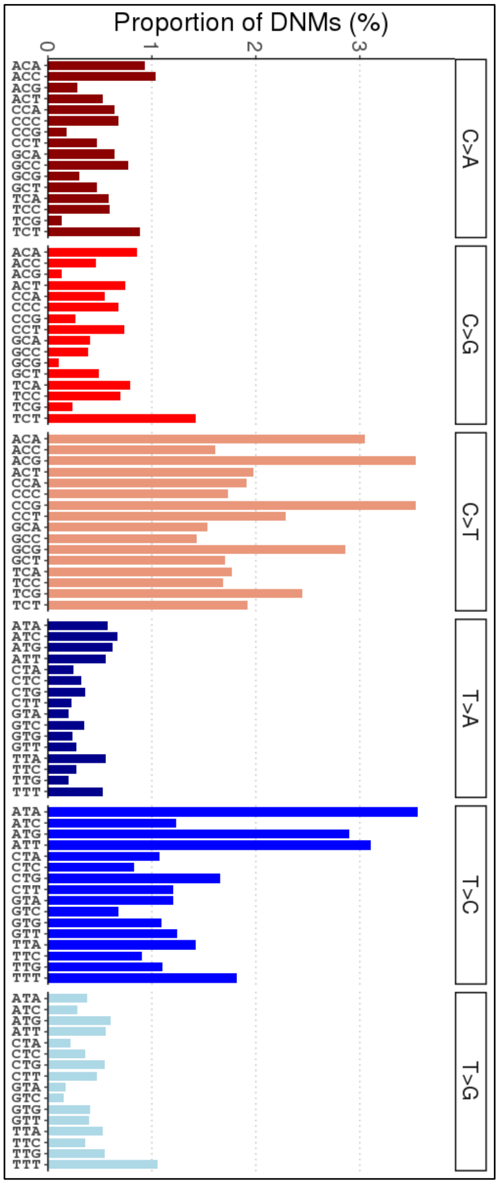

Supplementary Figure 8: Mutational signature of DNMs. A graphical representation of all the possible DNA sequence triplets at the DNM sites.

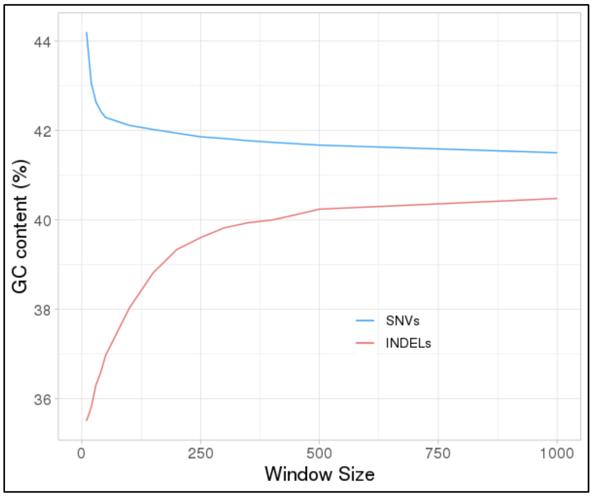

Supplementary Figure 9: GC content around DNMs. The blue (SNVs) and red (INDELs) lines show the percentage of G+C nucleotides in the DNA sequence around DNMs, using a sliding window that ranges from 10-1000 bases.

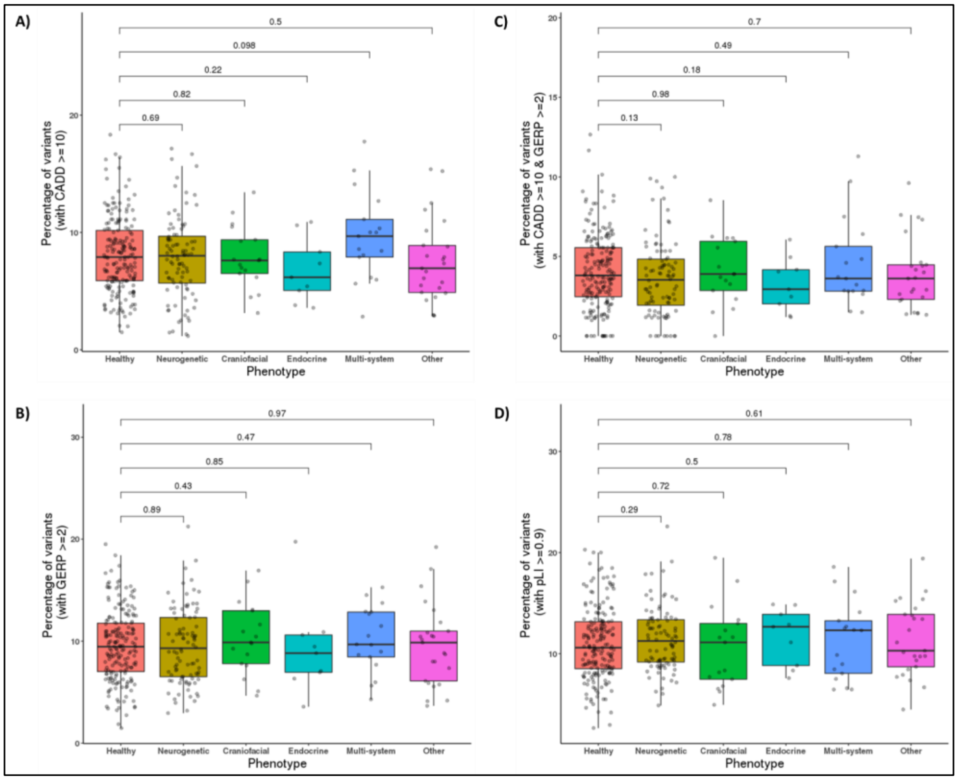

Supplementary Figure 10: Percentage of variants with different annotation metrics in different disease phenotypes. Boxplots show the median and interquartile range. For each phenotype, these boxplots show the percentage of variants with A) CADD score ≥10, B) GERP score ≥2, C) combined CADD score of ≥10 and GERP score of ≥2, and D) pLI score ≥0.9.

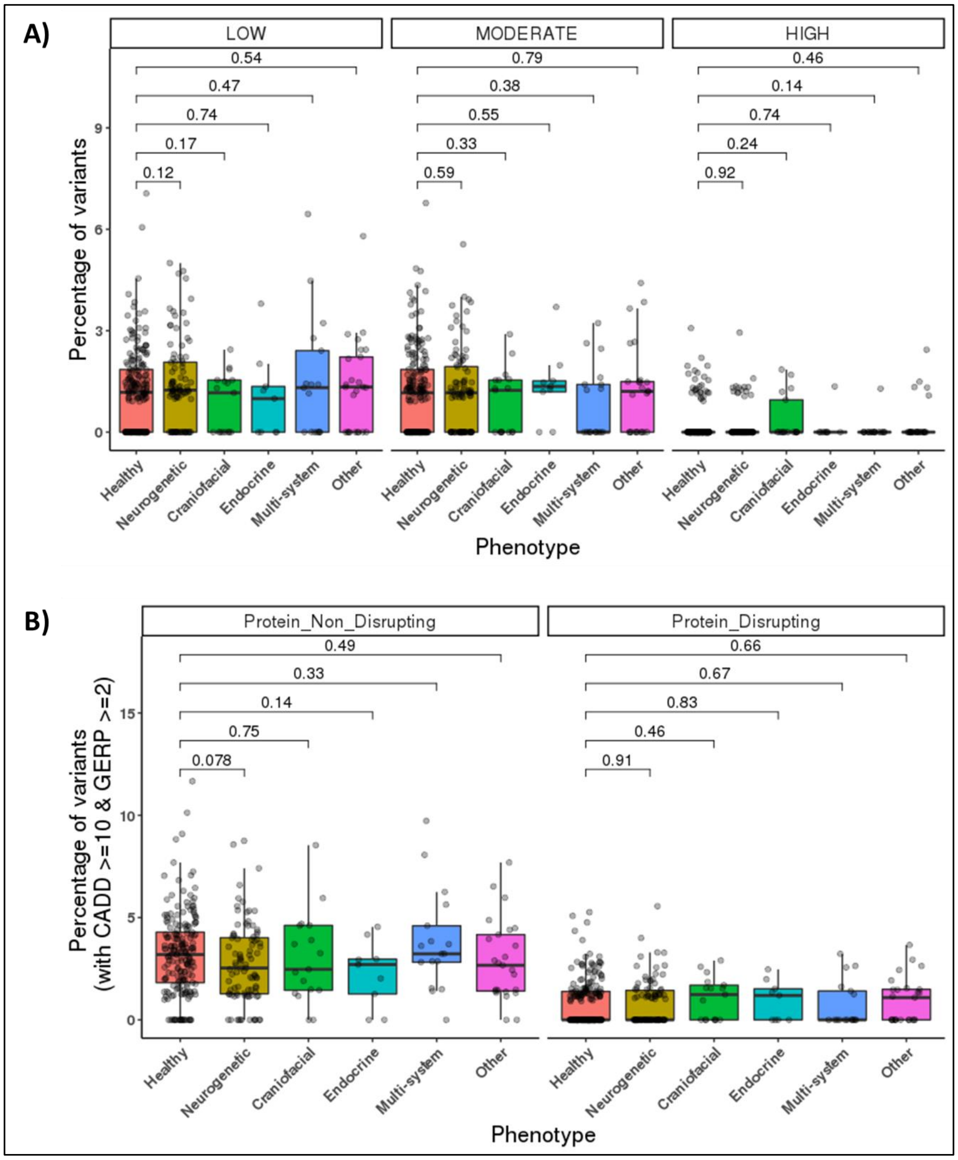

Supplementary Figure 11: Percentage of variants with regard to the functional impact in different disease phenotypes. Boxplots show the median and interquartile range. These boxplots show the percentage of variants in terms of the A) Functional impact, and B) Sub-categorized functional impact.

Supplementary Table 1: Poisson regression model for the effect of different factors on father age relationship with DNM counts (ANOVA test).

term(independent variable)	Resid.Df	Resid.Dev	Df	Deviance	Pr(>Chi)
father_age	103	151.87	1	120.59	<2.2e-16***
family_id	83	106.76	20	45.109	0.001067**
population	101	150.78	2	1.0848	0.5814
family_size	102	151.87	1	1.64E-05	0.9968
father_age:family_id	63	81.051	40	70.817	0.001905**
father_age:family_size	101	150.04	2	1.8331	0.3999

Formatted Table

Supplementary Table 2: Mutation rates of both transition and transversion variants with respect to CpG sites.

Mutation type	Count	Rate
Transition (CpG)	3 359	8.87×10^{-8}
Transition (Non-CpG)	12 914	6.67×10^{-9}
Transversion (CpG)	442	1.17×10^{-8}
Transversion (Non-CpG)	8 093	4.18×10^{-9}
**Total**	**24 808**	**1.26×10^{-8}**
