# Supplementary material for: Patterns and distribution of de novo mutations in multiplex Middle Eastern families
Source: J Hum Genet. 2022 Jun 20;67(10):579–88. doi: 10.1038/s10038-022-01054-9 (PMC9510050; doi:10.1038/s10038-022-01054-9)
Supplement: Supplementary file 2 [file 10038_2022_1054_MOESM2_ESM.pptx]

## Slide 1
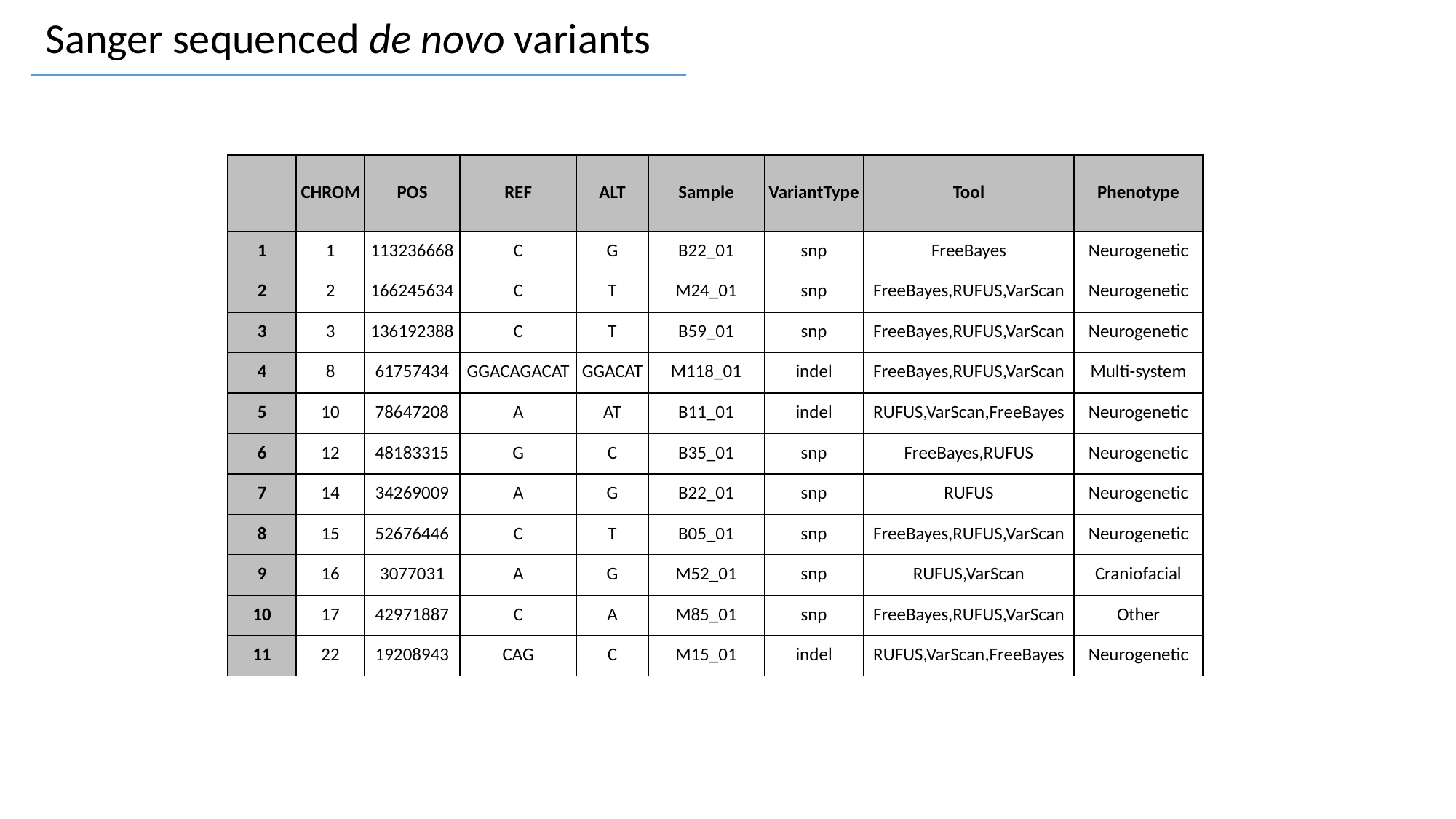

Sanger sequenced de novo variants
| | CHROM | POS | REF | ALT | Sample | VariantType | Tool | Phenotype |
| --- | --- | --- | --- | --- | --- | --- | --- | --- |
| 1 | 1 | 113236668 | C | G | B22\_01 | snp | FreeBayes | Neurogenetic |
| 2 | 2 | 166245634 | C | T | M24\_01 | snp | FreeBayes,RUFUS,VarScan | Neurogenetic |
| 3 | 3 | 136192388 | C | T | B59\_01 | snp | FreeBayes,RUFUS,VarScan | Neurogenetic |
| 4 | 8 | 61757434 | GGACAGACAT | GGACAT | M118\_01 | indel | FreeBayes,RUFUS,VarScan | Multi-system |
| 5 | 10 | 78647208 | A | AT | B11\_01 | indel | RUFUS,VarScan,FreeBayes | Neurogenetic |
| 6 | 12 | 48183315 | G | C | B35\_01 | snp | FreeBayes,RUFUS | Neurogenetic |
| 7 | 14 | 34269009 | A | G | B22\_01 | snp | RUFUS | Neurogenetic |
| 8 | 15 | 52676446 | C | T | B05\_01 | snp | FreeBayes,RUFUS,VarScan | Neurogenetic |
| 9 | 16 | 3077031 | A | G | M52\_01 | snp | RUFUS,VarScan | Craniofacial |
| 10 | 17 | 42971887 | C | A | M85\_01 | snp | FreeBayes,RUFUS,VarScan | Other |
| 11 | 22 | 19208943 | CAG | C | M15\_01 | indel | RUFUS,VarScan,FreeBayes | Neurogenetic |

## Slide 2
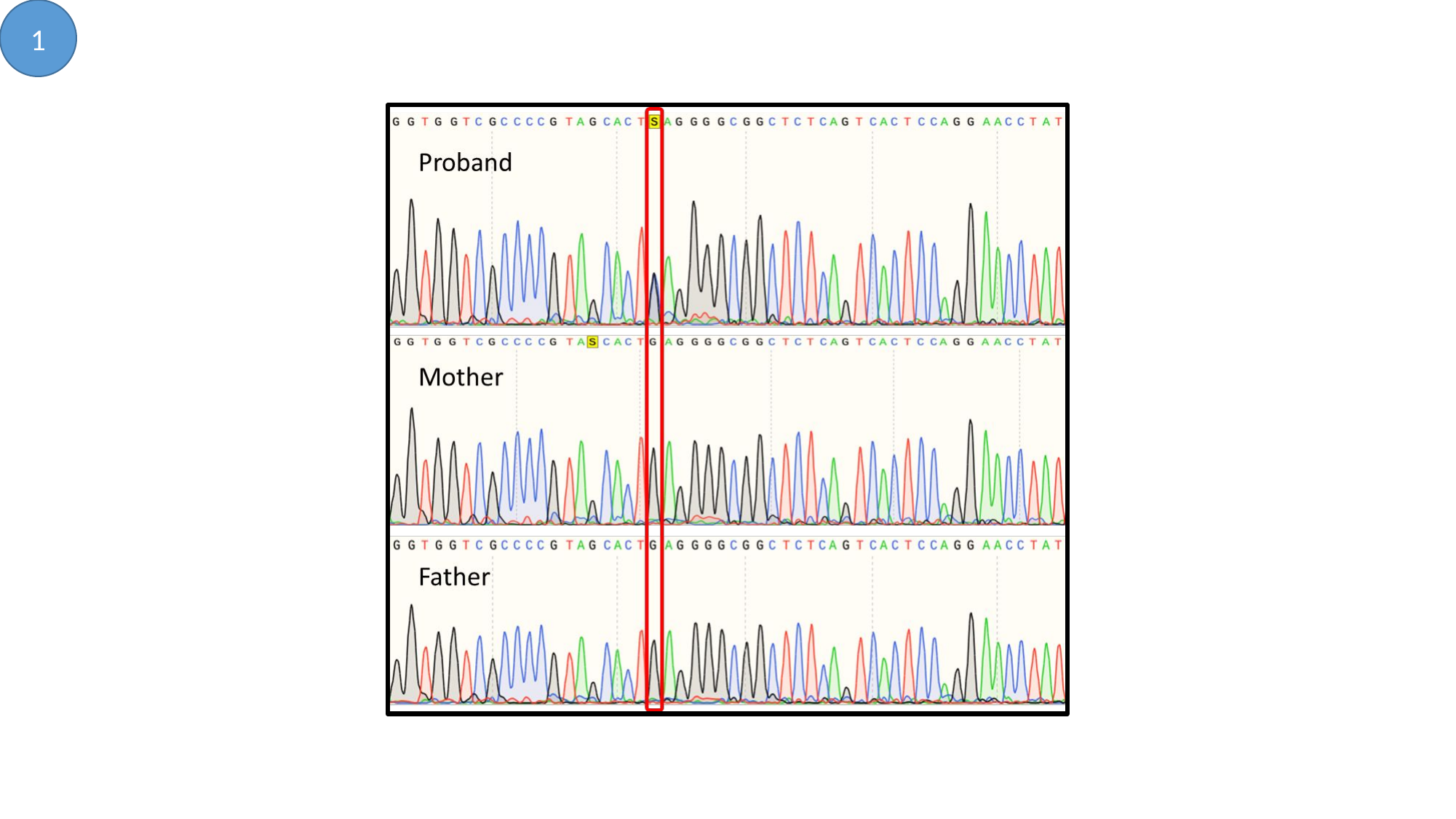

1

## Slide 3
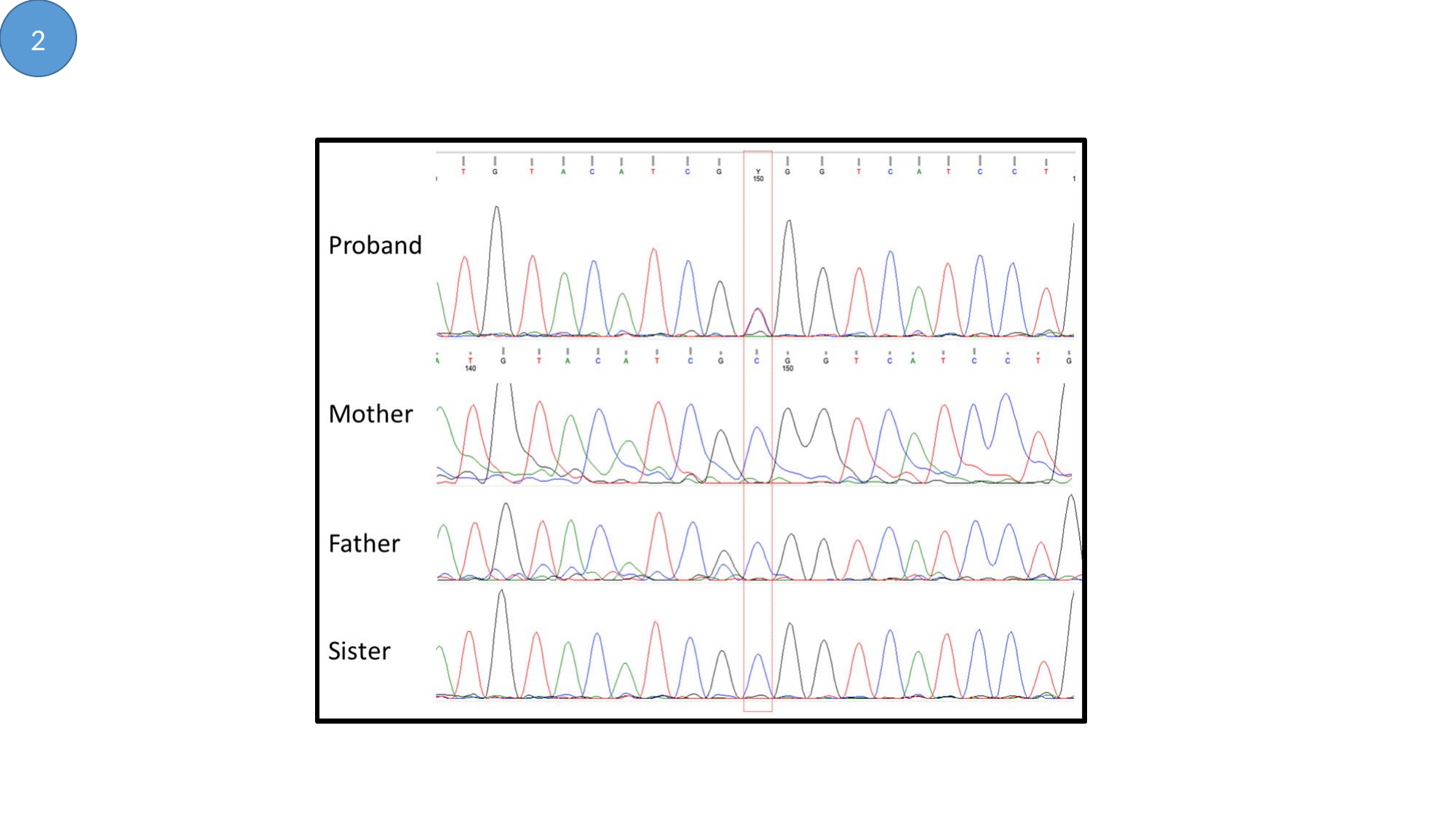

2

## Slide 4
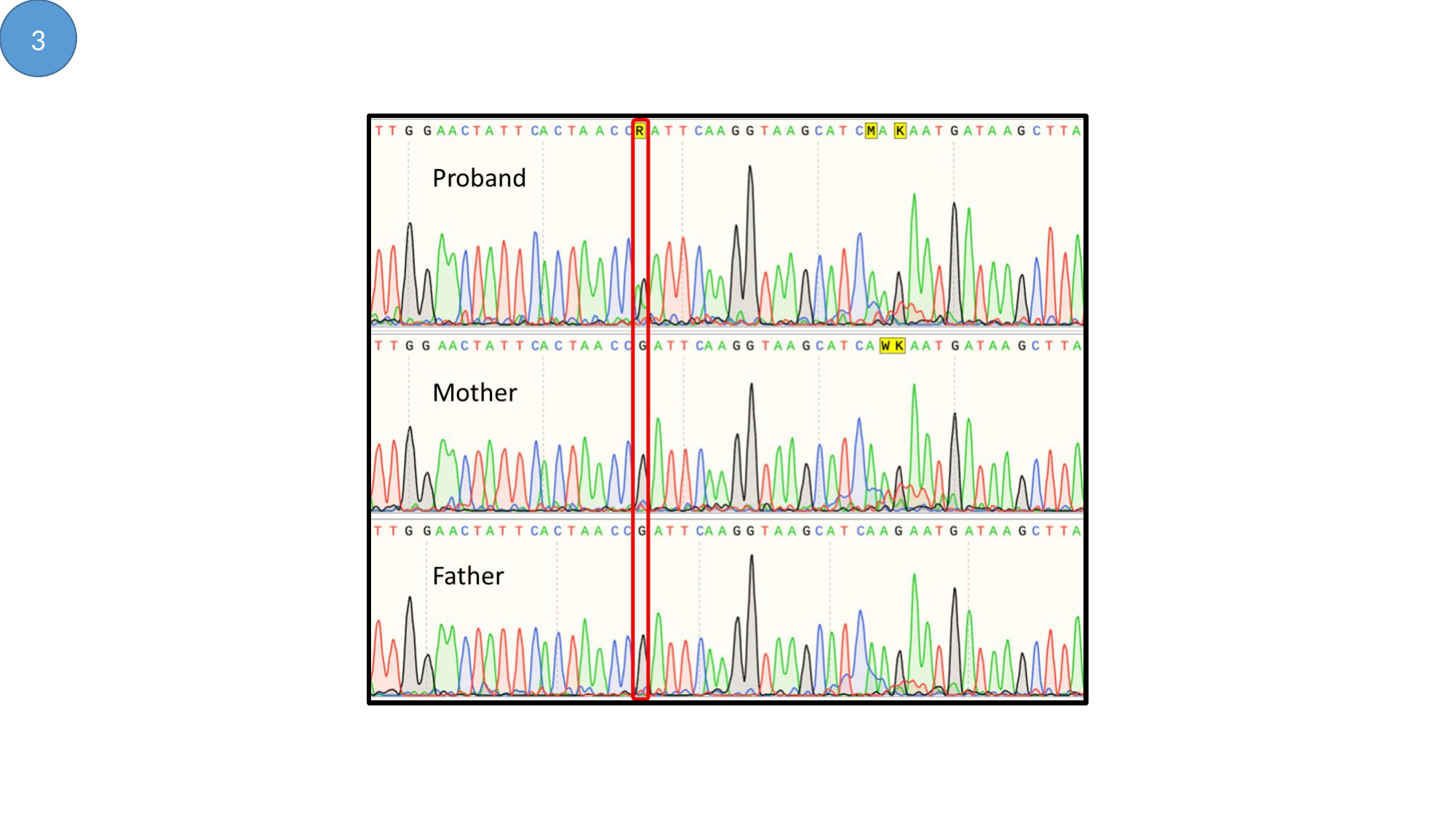

3

## Slide 5
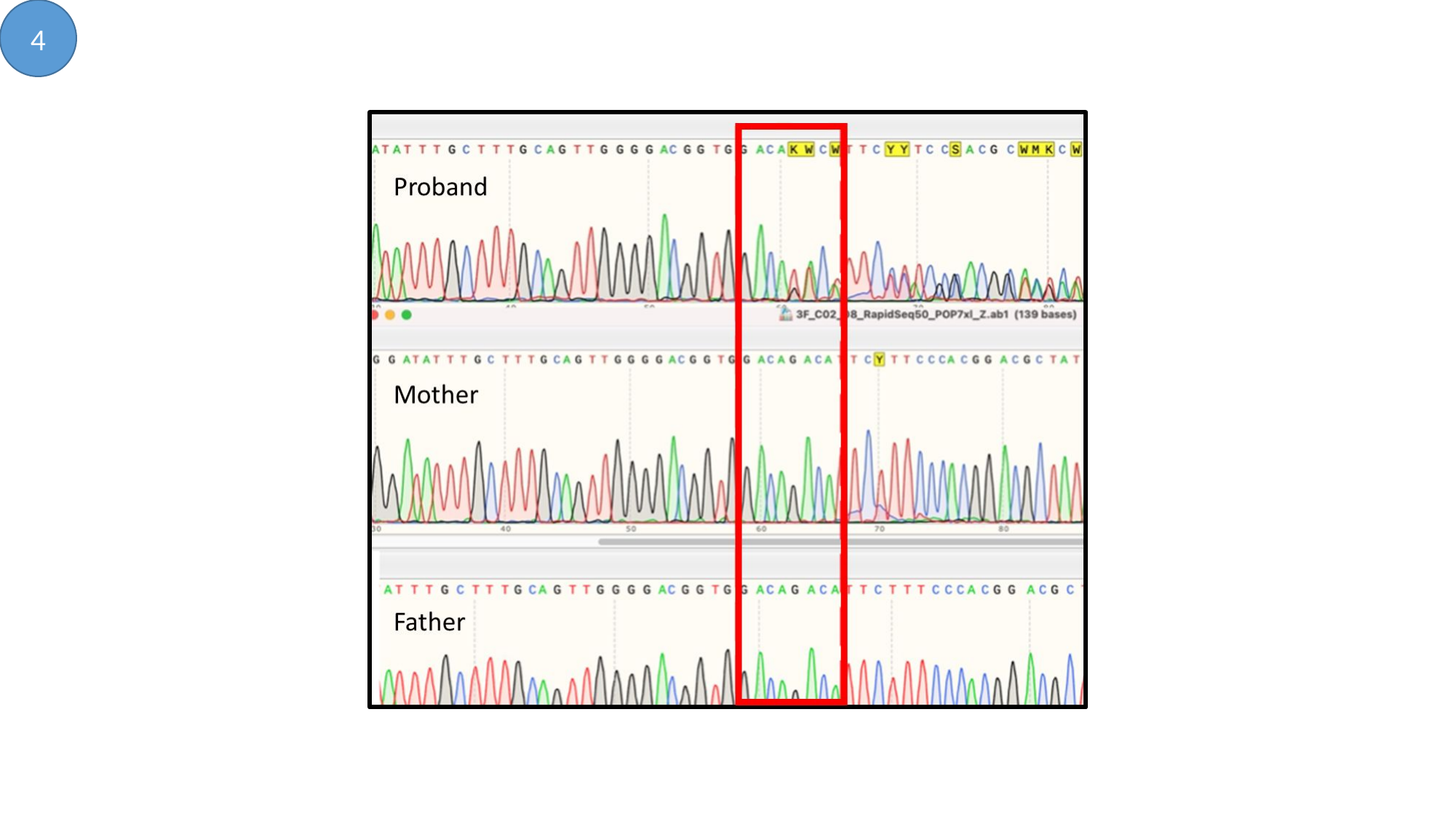

4

## Slide 6
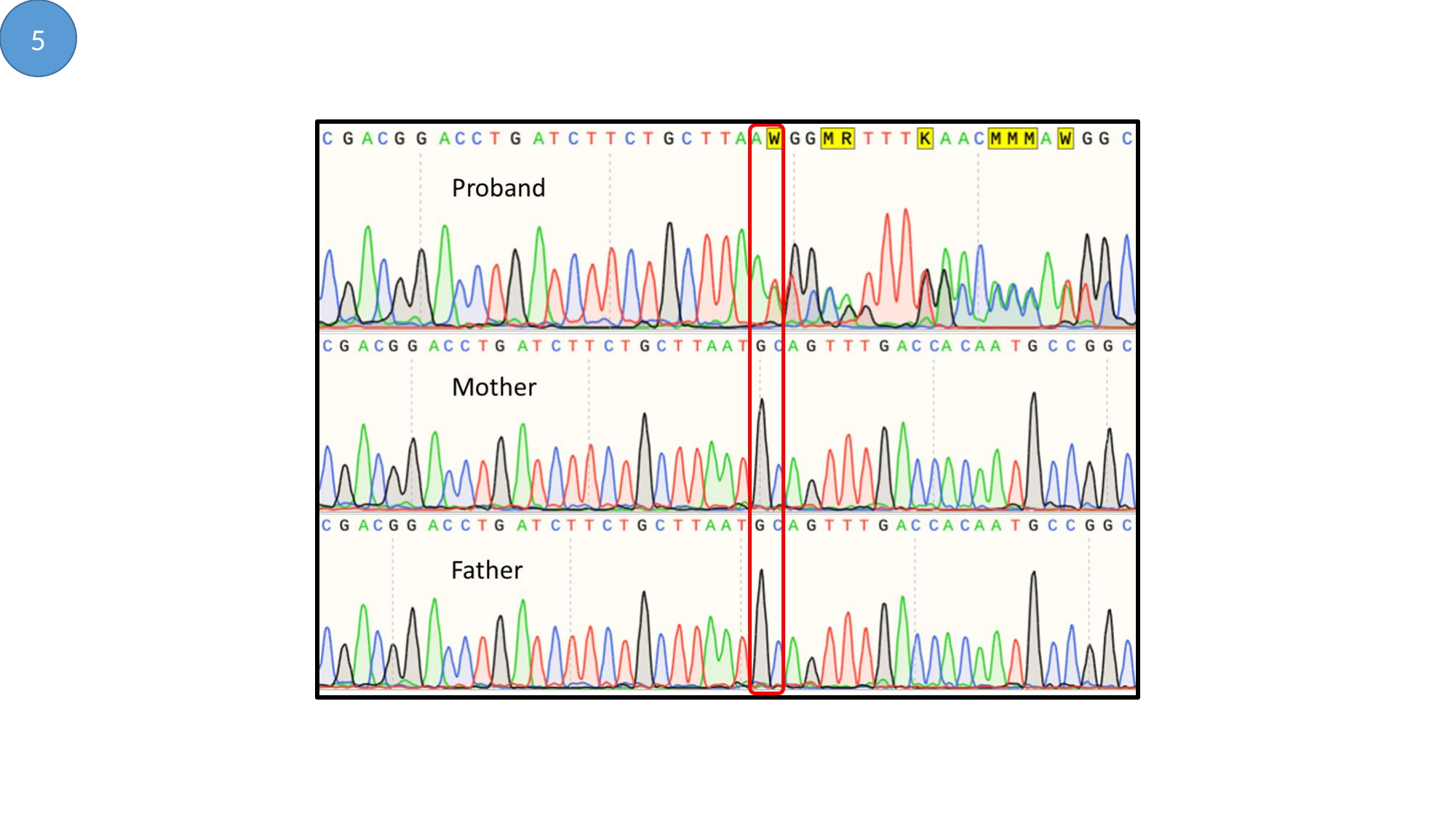

5

## Slide 7
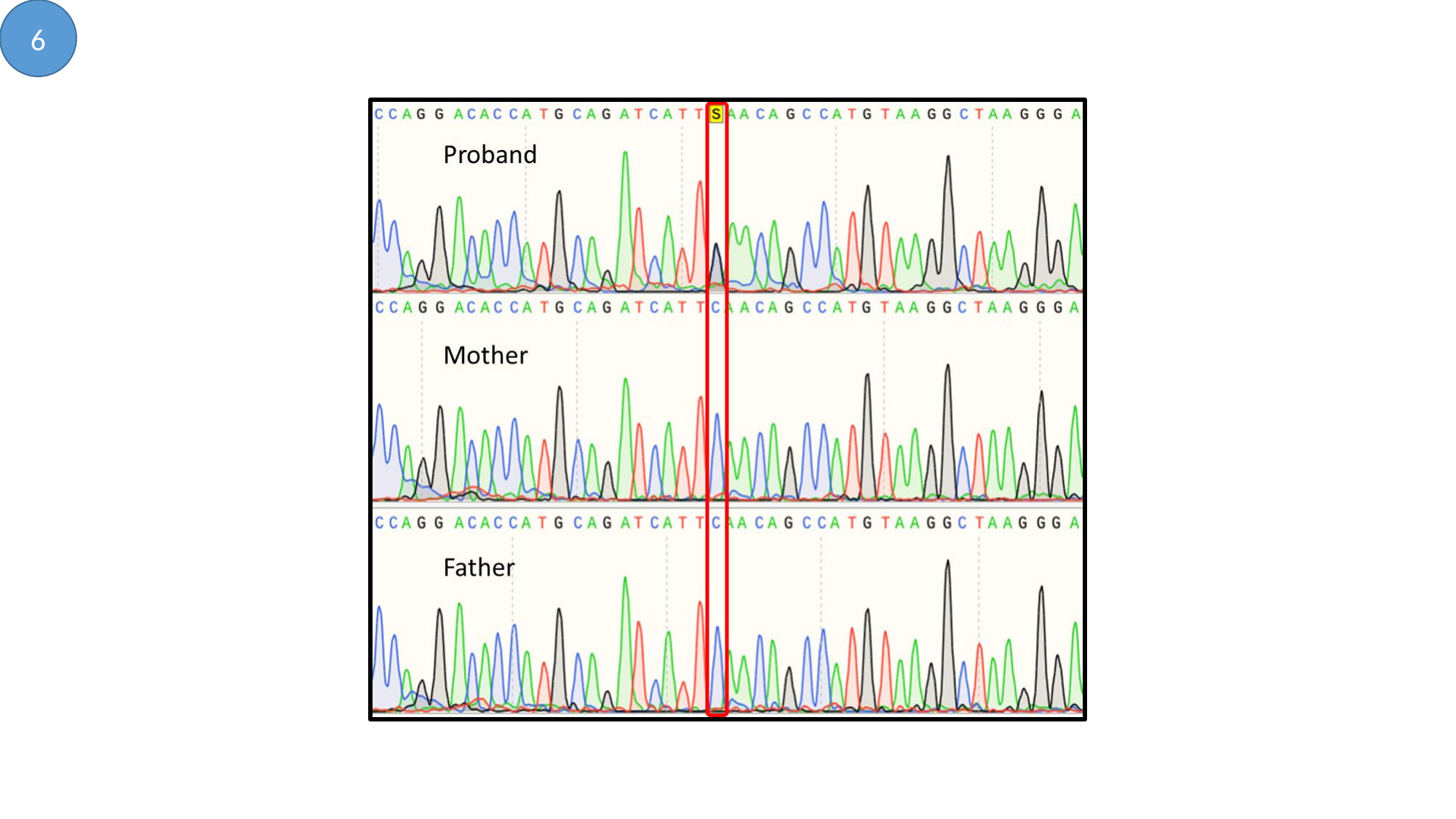

6

## Slide 8
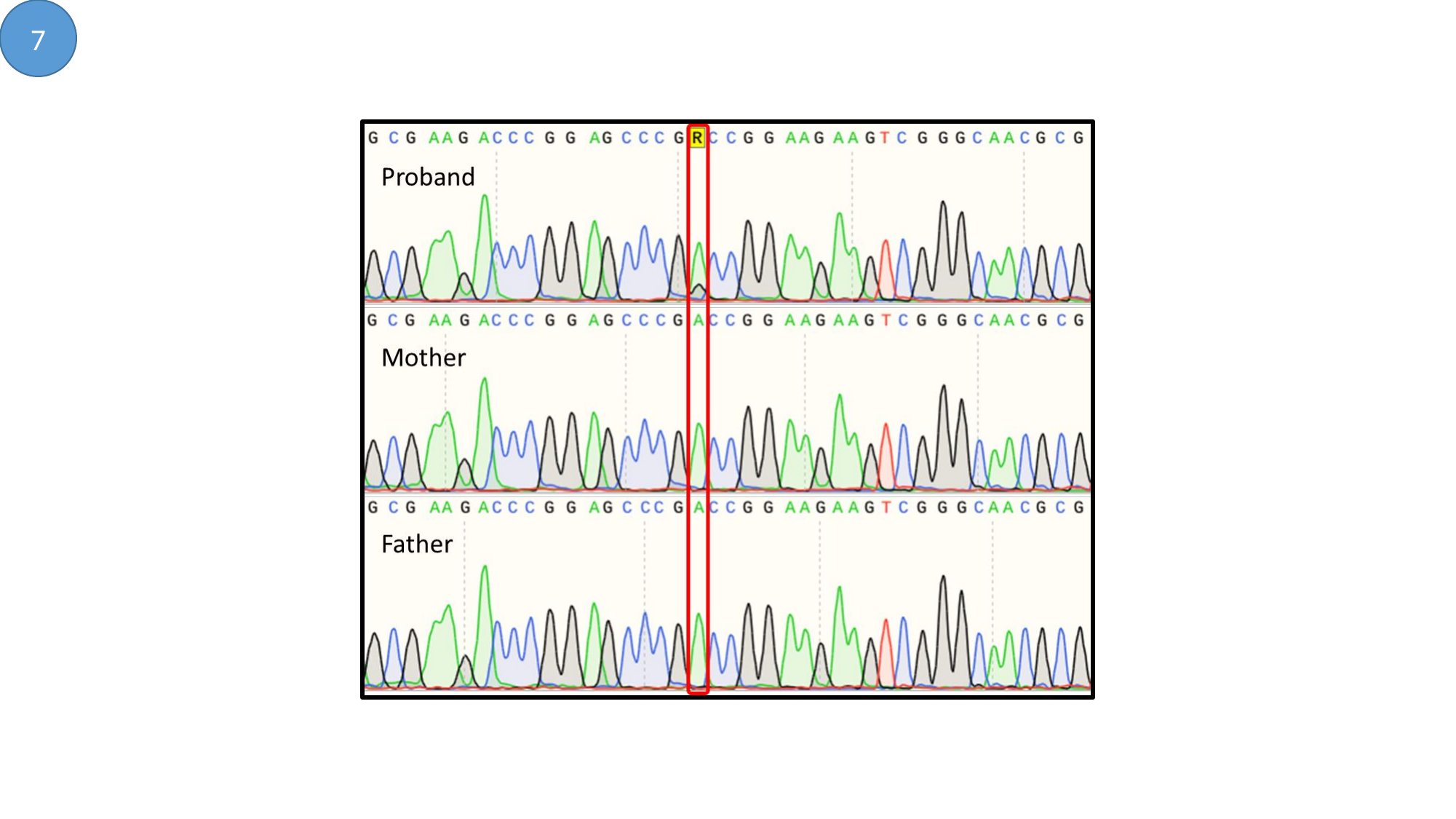

7

## Slide 9
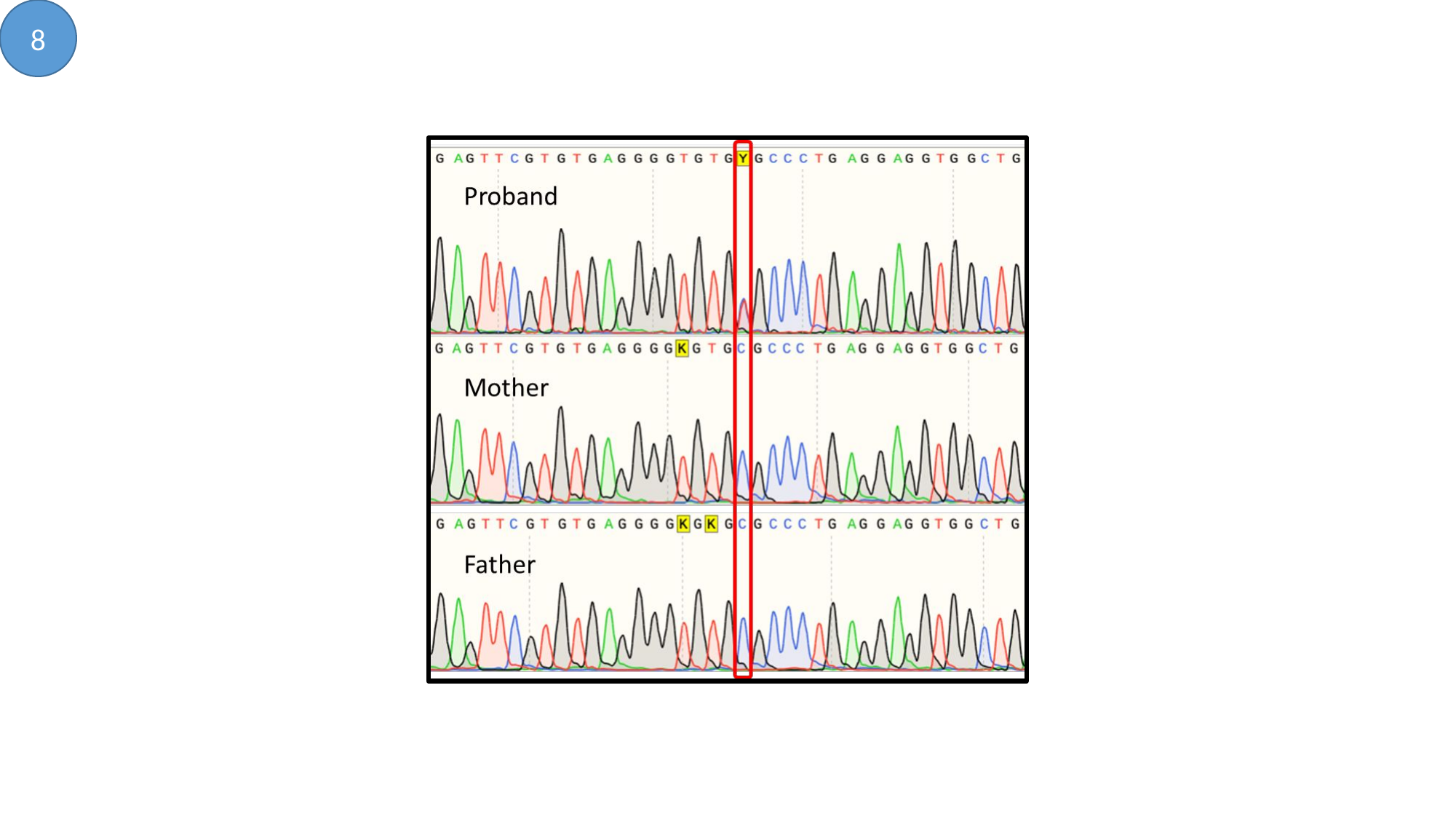

8

## Slide 10
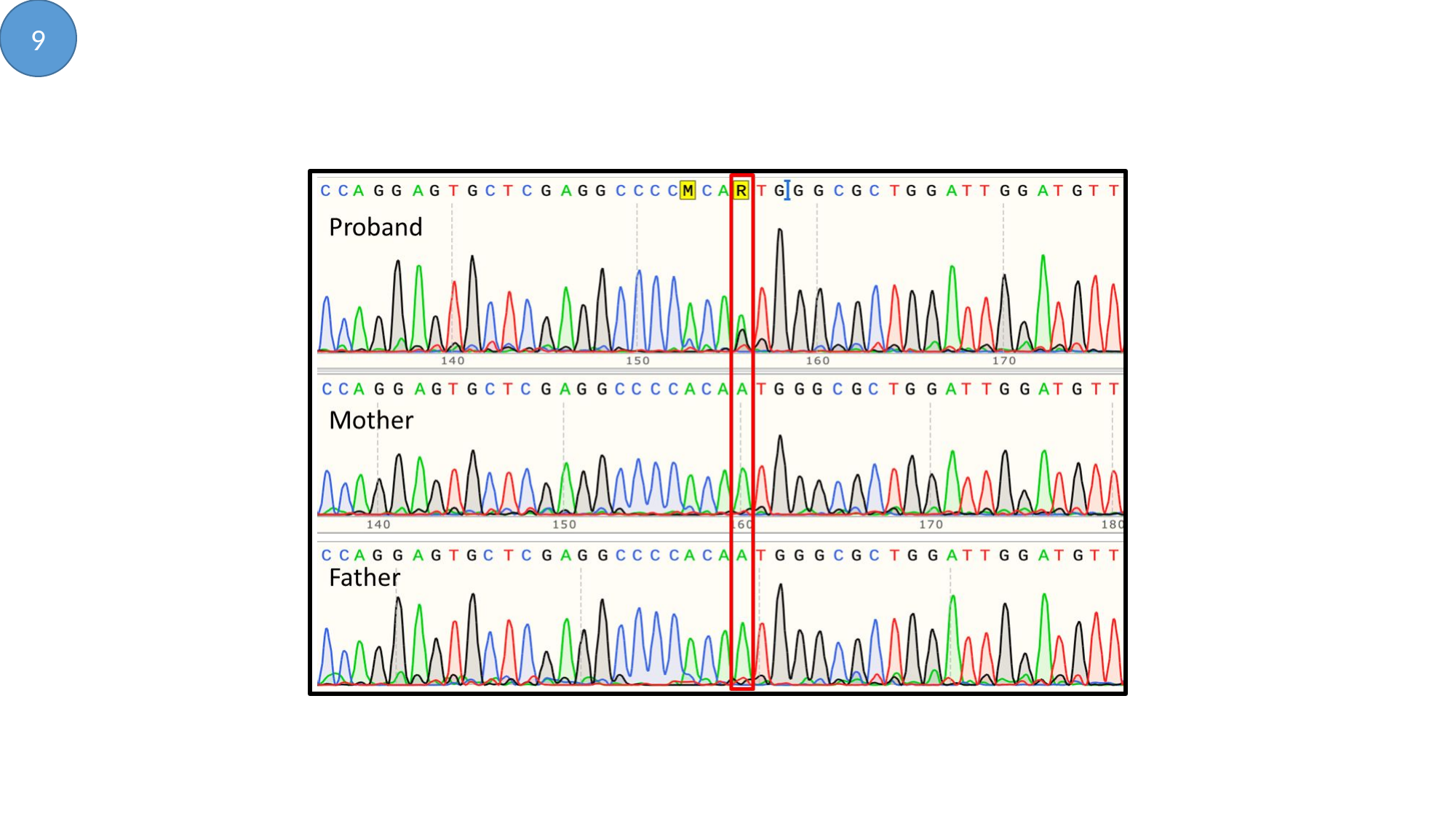

9

## Slide 11
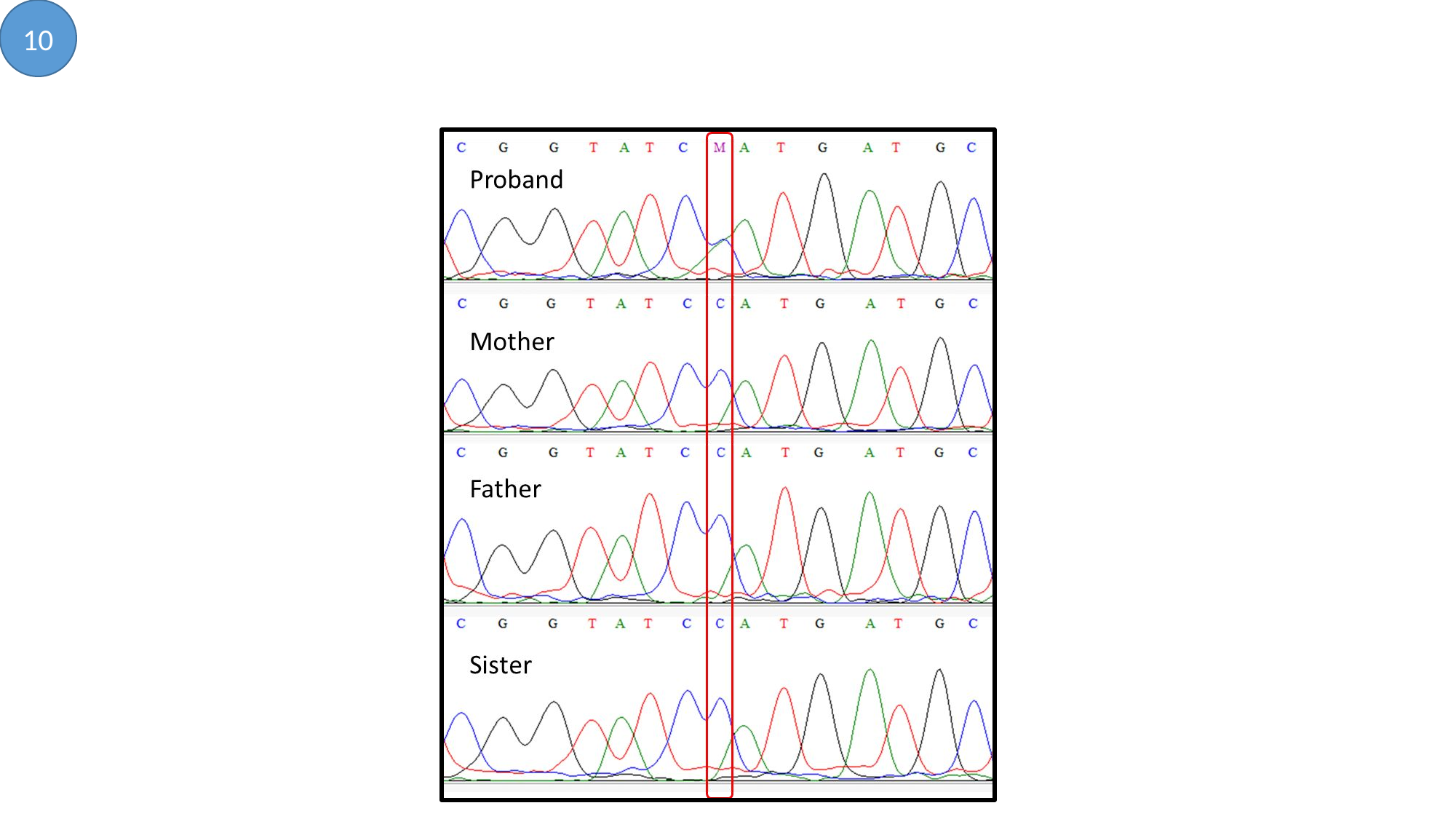

10

## Slide 12
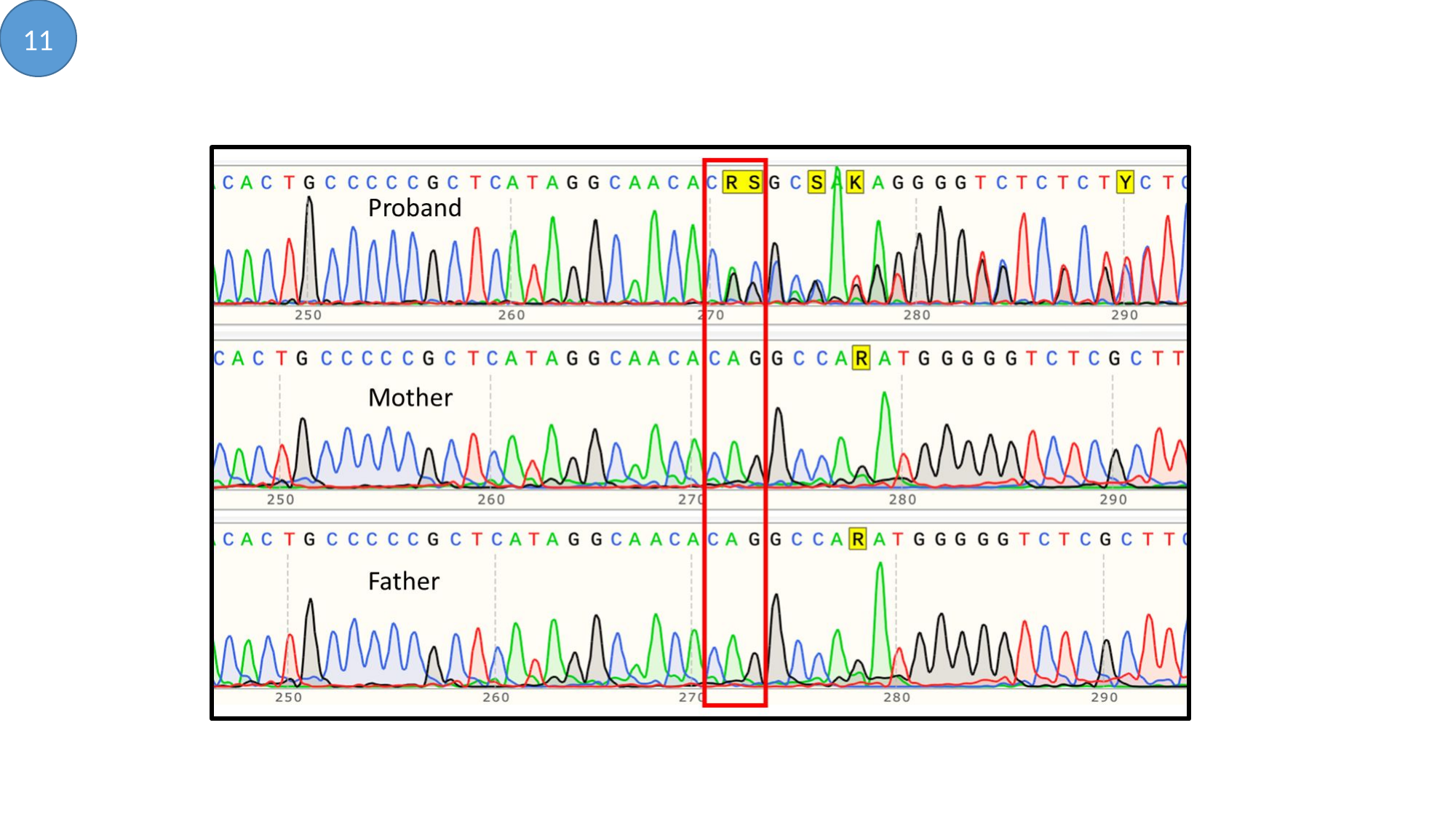

11
